# SNP rs11618521 modifies transcription factor NANOG-mediated expression of KPNA3 and the age at onset of spinocerebellar ataxia type 3

**DOI:** 10.1007/s00401-026-03051-0

**Published:** 2026-10-30

**Authors:** Hezhou Han, Leah Czisch, Manuel Pascal Wanner, Jonasz Jeremiasz Weber, Miriam Christina Dieterle, Carolina Alexandra Licia Schroeder, Pengfei Qi, Junbo Zhou, Jaqueline Jung, Wenxu Zheng, Fubo Cheng, Priscila Pereira Sena, Florian Fath, Gunnar Blumenstock, Carlos R. Gordon, Roy Zaltzman, Marcondes C. França, Maria-Luiza Saraiva-Pereira, Laura Bannach Jardim, Mario R. Cornejo-Olivas, Peter Bauer, Ludger Schöls, Bart P. C. van de Warrenburg, Alexandra Durr, Alexis Brice, Thomas Klockgether, Olaf Riess, The EUROSCA Network, Thorsten Schmidt

**Affiliations:** 1 Institute of Medical Genetics and Applied Genomics, University of Tübingen, Nägelestraße 5, 72076 Tübingen, Germany; 2 Department of Human Genetics, Ruhr University Bochum, 44801 Bochum, Germany; 3 Department of Clinical Epidemiology and Applied Biometry, University of Tübingen, 72076 Tübingen, Germany; 4 Department of Neurology, Meir Medical Center, Tel Aviv University, 69978 Tel Aviv, Israel; 5 Universidade Estadual de Campinas (UNICAMP), Campinas, 13083-970 Brazil; 6 Serviço de Genética Médica, Hospital de Clínicas de Porto Alegre, Porto Alegre, 90035-903 Brazil; 7 Departamento de Bioquímica, Universidade Federal Do Rio Grande Do Sul, Porto Alegre, 90035-003 Brazil; 8 Centros de Pesquisa Clínica E Experimental, Hospital de Clínicas de Porto Alegre, Porto Alegre, 90035‑903 Brazil; 9 Departamento de Medicina Interna, Faculdade de Medicina, Universidade Federal do Rio Grande do Sul, Porto Alegre, 90035-903 Brazil; 10 Neurogenetics Working Group, Universidad Científica del Sur, 15067 Lima, Peru; 11 Neurogenetics Research Center, Instituto Nacional de Ciencias Neurológicas, 15003 Lima, Peru; 12 Centogene GmbH, 18055 Rostock, Germany; 13 Pomeranian Medical University PUM, 70-204, Szczecin, Poland; 14 Department of Neurology and Hertie-Institute for Clinical Brain Research, University of Tübingen, 72076 Tübingen, Germany; 15 German Center of Neurodegenerative Diseases (DZNE), 72076 Tübingen, Germany; 16 Department of Neurology, Donders Institute for Brain, Cognition, and Behaviour, Radboud University Medical Center, 6525 Nijmegen, Netherlands; 17 Sorbonne Université, Institut du Cerveau – Paris Brain Institute – ICM, Inserm, CNRS, Assistance Publique Hôpitaux de Paris, Hôpital Pitié-Salpêtrière, Paris, France; 18 German Center for Neurodegenerative Diseases (DZNE), 53127 Bonn, Germany

**Keywords:** Machado–Joseph disease, Neurodegeneration, Karyopherin alpha 3, Importin alpha 4, Transcription factor, Polyglutamine (polyQ), Amcasertib

## Abstract

Intranuclear localization of the affected protein ataxin-3 is critical for the pathogenesis of spinocerebellar ataxia type 3 (SCA3). The nuclear–cytoplasmic transporter karyopherin alpha 3 (KPNA3) has been demonstrated to play an essential role in the pathophysiology of this disorder. In this study, we characterized the single nucleotide polymorphism (SNP) rs11618521, located in the *KPNA3* promoter, as a genetic modifier of the age at onset in SCA3 and a regulator of the *KPNA3* promoter activity. Moreover, we identified the transcription factor Nanog homeobox (NANOG) as a key regulator exerting allele-specific modulatory effects on the *KPNA3* promoter activity, thereby modulating expression and contributing to SCA3-associated pathogenic mechanisms. Pharmacological inhibition of NANOG reduced KPNA3 levels and ameliorated aggregate formation. In conclusion, we identified variant rs11618521 as a regulator of *KPNA3* promoter activity and as a genetic modifier of SCA3. Moreover, we provide mechanistic insights that may guide future-targeted therapeutic strategies.

**Supplementary Information:**

The online version contains supplementary material available at https://doi.org/10.1007/s00401-026-03051-0.

## Introduction

Spinocerebellar ataxia type 3 (SCA3; also known as Machado–Joseph disease, MJD) is an autosomal-dominant neurodegenerative disorder characterized by cerebellar ataxia, bulbar and pyramidal signs, extrapyramidal features, and, in some patients, peripheral neuropathy or ophthalmoplegia [48]. Although precise prevalence estimates for SCA3 vary by ethnicity and geography, this disorder is considered the most common polyglutamine (polyQ) ataxia worldwide, with an estimated global frequency of approximately 3 per 100,000 individuals [56]. Currently, there is no cure or disease-modifying therapy available for SCA3 [15].

SCA3 belongs to a group of polyglutamine expansion disorders, which are caused by pathogenic CAG trinucleotide repeat expansions within coding regions, leading to the production of proteins containing abnormally expanded polyQ tracts [63]. In SCA3, the CAG repeat expansion is located in exon 10 of the *ATXN3* gene, spanning approximately 12–44 repeats in the general population and 56–87 repeats in affected individuals [26, 36, 42]. The polyQ expansion drives a cascade of cellular dysfunctions, including aberrant proteolysis, mitochondrial impairment, autophagy disruption, and pathological protein aggregation [34, 60].

Intranuclear localization and aggregation of polyQ-expanded ataxin-3 play a critical role in the pathophysiology of SCA3 [7]. Moreover, we have previously identified the nuclear transport protein karyopherin α-3 (KPNA3, also known as importin α-4; Gene ID 3839) as a crucial regulator of ataxin-3 subcellular localization [51]. In vitro and in vivo, studies demonstrated that KPNA3 controls ataxin-3 localization, aggregation, and toxicity. Notably, *KPNA3* knockdown or knockout in *Drosophila* and mouse models of SCA3 mitigated neurological phenotypes induced by polyQ-expanded ataxin-3 [51]. These observations highlight *KPNA3* as a potential genetic modifier and motivate a deeper investigation of its regulation in SCA3.

The age at onset (AAO) of SCA3 ranges widely, from 5 to 75 years (mean approximately 30–40 years), and is inversely correlated with the CAG repeat length [31]. Although the repeat length is the primary determinant of the AAO, considerable variability remains unexplained, suggesting the contribution of additional genetic or environmental modifiers [31, 56]. Several genetic factors have been identified as AAO modifiers in SCA3, including single nucleotide polymorphisms (SNPs) in genes such as *RAG*, *TRIM29*, *PRKN* and *APOE*, as well as non-pathological CAG repeat lengths at *ATXN2*, *ATN1* and *HTT* [1, 6, 18, 37, 53, 58]. Given the critical role of *KPNA3* in regulating SCA3 pathogenesis, genetic variants that influence its expression may likewise modify disease onset and progression. However, such regulatory variants have not been systematically characterized.

Promoter variants are well-recognized contributors to cis-regulatory variation and can substantially affect gene expression [3, 11, 20]. Mechanistically, promoters initiate transcription by assembling the pre-initiation complex, including general transcription factors (TF) and RNA polymerase II, under the guidance of sequence-specific TF. TF-promoter binding is dictated by short DNA motifs whose affinities are highly sensitive to single nucleotide changes. Accordingly, promoter variants can increase or decrease TF occupancy [29]. Therefore, SNPs located within promoters may influence disease phenotypes by altering gene expression.

In this study, we characterized the SNP rs11618521, located in the *KPNA3* promoter, for its potential to affect *KPNA3* expression and to modify SCA3. We performed genotype–AAO correlation analyses in a large SCA3 patient cohort and combined these with cell-based functional assays to elucidate and validate the mechanism by which rs11618521 could regulate *KPNA3* expression. Taken together, our findings demonstrated that this *KPNA3* promoter variant contributes to the molecular pathogenesis of SCA3 and acts as a genetic modifier influencing disease onset.

## Materials and methods

### DNA samples from SCA3 patients

The SCA3 patient cohorts included in this study were collected within the EUROSCA [47] and EuSAge projects. Cohort characteristics are summarized in Table 1. Genomic DNA was amplified using the REPLI-g method (150035, QIAGEN, Hilden, Germany) and diluted at a 3:200 ratio prior to downstream analyses. Use of these samples was approved by the Ethics Committee of the University of Tübingen, Germany (Processing number 046/2013B02). All patients provided written informed consent for participation and publication of the data.

**Table 1:** Mean unadjusted and adjusted age at onset per *KPNA3* SNP rs11618521 genotype, and analysis of differences in CAG repeat numbers and the age at onset between genotypes in the SCA3 cohort

Genotype	Patients, *n*	CAG repeats [range]	AAO, years [range]	AAO-adjusted, years
C/C	184	70.7 ± 0.4 [57–81]	38.4 ± 0.9 [10–74]	38.8 ± 0.7
C/T	203	70.6 ± 0.3 [56–82]	41.7 ± 0.8 [15–73]	42.0 ± 0.7
T/T	77	71.2 ± 0.5 [58–81]	40.5 ± 1.4 [17–78]	41.6 ± 1.0
All combined	464	70.8 ± 0.2 [56–82]	40.2 ± 0.6 [10–78]	
Kruskal–Wallis test, *p*		0.52	0.029	

We observed a statistically significant impact of the SNP on the age at onset (AAO). After adjustment of the mean AAO to account for minor differences among genotype groups, patients homozygous for the major allele (C/C) have an approximately 3-year earlier mean AAO than carriers of the minor allele T

### Cell lines

HEK293T cells (ATCC ID: CRL-3216), U87-MG cells (ATCC ID: HTB-14), SH-SY5Y cells (ATCC ID: CRL-2266), SK-N-AS cells (ATCC ID: CRL-2137) and immortalized SCA3 human fibroblast [59] were cultured in Dulbecco’s modified Eagle’s medium (DMEM, 10566016, Thermo Fisher Scientific) supplemented with 10% fetal bovine serum (FBS, 10099141C, Thermo Fisher Scientific), 1% non-essential amino acids (NEAA, 11140050, Thermo Fisher Scientific), and 1% antibiotic–antimycotic (A/A, 15240062, Thermo Fisher Scientific) in 5% CO_2_ at 37 °C.

### Mouse samples

C57BL/6N mouse brain tissues were obtained and regionally dissected at the Institute of Medical Genetics and Applied Genomics, University of Tübingen. All procedures were approved by Regierungspräsidum Tübingen (License Number: HG02/21G) and carried out in accordance with the German Animal Welfare Act and the guidelines of the Federation of European Laboratory Animal Science Associations, based on European Union legislation (Directive 2010/63/EU).

### DNA isolation from cells

Genomic DNA from individual cell lines was extracted using the high pure PCR template preparation kit (11796828001, Roche Diagnostics, Mannheim, Germany) according to the manufacturer’s instructions. Briefly, 5 × 10^5^ cells of each cell line were harvested, washed with Dulbecco’s phosphate-buffered saline (DPBS, 10010023, Thermo Fisher Scientific) and lysed with binding buffer plus proteinase K at 70 °C for 10 min. Lysates were mixed with isopropanol, applied to the silica column, washed (inhibitor removal buffer and wash buffers I/II), and DNA was eluted with 200 µl elution buffer prewarmed to 70 °C. DNA concentration and purity were determined spectrophotometrically at A260/A280 by a BioPhotameter (Eppendorf, Hamburg, Germany). Extracted DNA was stored at - 20 °C and used for subsequent PCR amplification and genotyping.

### PCR

PCR primers were generated using Primer 3 (http://primer3.ut.ee/). For rs11618521 genotyping, the assay with the primer pair *KPNA3*-forward (5′–3′: GGAGGGCAGATCACAAGG) and *KPNA3*-reverse (5′–3′: ACATACCTGCTCATGTCTACCA) amplified a 338 bp long PCR product. The PCR reaction mixture and cycling protocol are detailed in Supplementary Table S1 and Supplementary Table S2.

### High-resolution melting analysis

High-resolution melting (HRM) is a method for detecting sequence polymorphisms and assigning genotypes. After PCR amplification of the target gene segments, the double-stranded PCR products are gradually denatured, and their melting behavior is recorded to generate melting curves. Genotypes produce characteristic melting profiles, enabling patient genotyping. For HRM, we used the LightCycler 480 High Resolution Melting Master (04909631001, Roche Diagnostics). PCR amplification and subsequent melting were performed on a Roche LightCycler 480 using the gene scanning 384 program. Melting curves were evaluated with LightCycler 480 software version 1.5.0 SP3 (Roche Diagnostics, Mannheim, Germany).

### Sanger sequencing

To corroborate the variants detected by high-resolution melt profiling, we performed Sanger sequencing. PCR amplicons were first purified with QIAquick PCR purification columns (28106, QIAGEN) in accordance with the supplier’s protocol. Dye-terminator cycle sequencing was then separated on a CEQ 8000 genetic analysis system (Beckman/AB SCIEX, Krefeld, Germany) following the GenomeLab quick start guidelines (608120, Beckman Coulter).

### Identification of candidate transcription factors

We searched for candidate transcription factors (TFs) by using publicly available databases (Supplementary Table S3). Specifically, predicted TFs were identified by comparing in silico allele-specific binding predictions for *KPNA3* promoter sequences harboring the C or T allele of rs11618521, whereas experimentally supported data on TF were extracted from public ChIP-based datasets reporting occupancy at or near the rs11618521 locus. Detailed information on TF expression constructs can be found in Supplementary Table S4.

### Cell transfection and treatment

For transfection, cells were seeded at an appropriate density to reach 70–80% confluency before transfection. For overexpression, Turbofectin 8.0 (TF81001, Origene Technologies, Rockville, MD, USA) was used according to the manufacturer’s instructions. For gene knockdown, HEK293T and U87-MG cells were transfected with esiRNA using Effectene (301425, QIAGEN) for HEK293T and Attractene (301005, QIAGEN) for U87-MG cells, following the manufacturer’s protocols with a final esiRNA concentration of 20 nM. esiRNAs were all obtained from Sigma-Aldrich Chemie (Taufkirchen, Germany). Cells were maintained at 37 °C, 5% CO_2_ in a humidified incubator until analysis. For Amcasertib (HY-17602, Med ChemExpress, Sollentuna, Sweden) treatment, Amcasertib was first dissolved in dimethyl sulfoxide (DMSO, A3006-0100, AppliChem, Darmstadt, Germany) to prepare a stock solution (1 mM). Prior to cell treatment, the stock solution was diluted with Opti-MEM (31985070, Thermo Fisher Scientific) to the desired working concentration and subsequently added to the cell culture medium. The final concentration of DMSO in all treatments was maintained below 0.1% to avoid solvent-induced cytotoxicity.

### Dual-luciferase assay

Reporter assays were performed in 384-well microplates. 5000 cells per well were seeded in complete medium and co-transfected with or without a TF expression plasmid, a promoter–reporter construct harboring the KPNA3 promoter region upstream of the firefly luciferase gene (pGL3-Vector) and a *Renilla* control (pRL-CMV) using Turbofectin 8.0 following the manufacturer’s instructions. DNA and reagent were mixed in Opti-MEM, incubated to allow complex formation, and added to wells. Cells were maintained under standard culture conditions (37 °C, 5% CO_2_) until assay. Luciferase activity was measured using the Dual-Glo luciferase assay system (E2920, Promega, Walldorf, Germany) in a MicroLumat Plus LB96V luminometer (Berthold Technologies, Bad Wildbad, Germany). Results were analyzed as the firefly/*Renilla* ratio.

### Chromatin immunoprecipitation PCR

Chromatin immunoprecipitation (ChIP) was performed as previously reported [13]. Briefly, 2 million cells were crosslinked with 1% formaldehyde and sonicated in RIPA buffer to obtain chromatin fragments of 200–500 bp. The chromatin was incubated with rabbit anti-NANOG antibody (14295–1-AP, Proteintech, Planegg-Martinsried, Germany) or normal rabbit IgG (2729S, Cell Signaling Technology, Leiden, The Netherlands), which had been pre-incubated with Dynabeads Protein G beads (10003D, Thermo Fisher Scientific). Normal rabbit IgG was used as a negative control. After washing, the precipitated immunocomplexes were treated with proteinase K, and DNA was purified using the QIAquick PCR Purification Kit (28106, QIAGEN). The ChIP-eluted DNA was used for PCR analysis. The utilized primers were *KPNA3*-promoter-forward (5′–3′: ACAAGCAAATGAAGCCCACC) and *KPNA3*-promoter-reverse (5′–3′: TCAAGAGACCGAGACCAGTC). The PCR reaction mixture and cycling protocol are detailed in Table S1 and Table S2.

### Agarose gel electrophoresis

Agarose gel electrophoresis was performed using agarose dissolved in TAE buffer (40 mM Tris–acetate, 1 mM EDTA pH 8.0). After heating and complete dissolution, ethidium bromide was added, and the gel was cast and allowed to solidify at room temperature. DNA samples mixed with loading buffer were loaded into the wells, and electrophoresis was conducted in TAE buffer under constant voltage. DNA bands were subsequently visualized using UVP Gel Studio PLUS (Analytik Jena, Jena, Germany).

### Cell viability assay

5000 cells per well were plated in black 96-well microplates. 24 h later, cells were treated with different concentrations of Amcasertib and maintained for an additional 48 h. After treatment, the medium was removed and replaced with fresh medium containing PrestoBlue cell viability reagent (A13261, Thermo Fisher Scientific) at a 1:10 dilution. Plates were incubated for 10 min under standard growth conditions, and fluorescence was recorded with a Synergy HT plate reader (BioTek Instruments, Winooski, VT, USA) using an excitation wavelength of 535 nm and emission at 615 nm and analyzed via Gen5 software.

### Protein extraction

HEK293T cells were harvested by gentle PBS washing, whereas the other cell lines were detached with TrypLE (12605010, Gibco) and collected into 1.5 ml microcentrifuge tubes. After centrifugation at 1000×*g* for 10 min, the supernatant was discarded and the pellet was rinsed once with ice-cold DPBS. The cell pellet was then resuspended in ice-cold RIPA lysis buffer (50 mM Tris–HCl pH 7.5, 150 mM NaCl, 1% Triton X-100, 0.5% sodium deoxycholate, 0.1% SDS) supplemented with cOmplete protease inhibitor cocktail (11836170001, Roche). Cells were disrupted with a Sonopuls probe sonicator (Bandelin, Berlin, Germany) using a single 3 s burst at 10% amplitude (10% duty cycle). The resulting homogenate was incubated on ice for 15 min with vortexing every 5 min. A portion of the homogenate was reserved for the filter retardation assay, while the remaining lysate was clarified by centrifugation at 13,400×*g* for 15 min at 4 °C. The resulting supernatant was transferred to pre-cooled tubes. Protein concentrations were determined in a microplate format using the Bradford method (5000002, Bio-Rad Laboratories, Hercules, CA, USA).

### Western blotting

Protein lysates were mixed with 4 × LDS loading buffer (1 M Tris Base pH 8.5, 2 mM EDTA, 8% LDS, 40% glycerol, 0.1% Orange G) at a 3:1 ratio, supplemented with 100 mM dithiothreitol, and denatured for 10 min at 70 °C. Samples were separated on Bis–Tris polyacrylamide gels in MOPS running buffer (50 mM MOPS, 50 mM Tris–HCl pH 7.7, 0.1% SDS, 1 mM EDTA). Proteins were then transferred to Amersham Protran Premium nitrocellulose membranes (0.2 µm; Cytiva, Freiburg, Germany) in a TE22 transfer tank (Hoefer, Freiburg, Germany) at 80 V with a current limit of 250 mA for 2 h in Bicine/Bis–Tris buffer (25 mM Bicine, 25 mM Bis–Tris pH 7.2, 1 mM EDTA, 15% Methanol). If total protein loading needed to be assessed as an internal control, SYPRO ruby protein blot stain (S11791, Thermo Fisher Scientific) was used following the protocol. In brief, this involves staining the membrane with SYPRO ruby protein blot stain, washing to remove excess dye, and then imaging the membrane on a LI-COR ODYSSEY FC imager and quantifying using Image Studio 4.0 software (both LI-COR Biosciences, Lincoln, NE, USA). Membranes were then blocked for 1 h in 5% skim milk powder (70166, Merck) prepared in TBS (10 mM Tris–HCl, 150 mM NaCl, pH 7.5) and washed 15 min. Then, the membranes were incubated overnight at 4 °C with primary antibodies diluted in TBS-T (TBS + 0.1% Tween-20). After three washes in TBS-T, membranes were treated for 2 h at room temperature with respective secondary antibodies. The antibodies used in this study can be found in Supplementary Table S5. The blots were imaged after the washing of 15 min with TBS-T.

### Filter retardation assay

To analyze SDS-insoluble protein species, 500 ng of each cell homogenate was diluted in DPBS supplemented with 2% SDS and 50 mM dithiothreitol, then boiled at 95 °C for 5 min. Denatured samples were applied to an Amersham Protran nitrocellulose membrane (0.45 µm; Cytiva) using a Minifold II slot-blot apparatus (Schleicher & Schuell, Dassel, Germany). Membranes were blocked in 5% skim milk powder prepared in TBS and probed with the indicated primary and IRDye-conjugated secondary antibodies. Fluorescent signals were captured on a LI-COR ODYSSEY FC imager and quantified with Image Studio 4.0 software.

### Microscopy

For fluorescence imaging of cells, cells were seeded on poly-L-lysine-coated 24-well plates or sterile glass coverslips. After 24 h, cultures were transfected with the indicated pEGFP-ataxin-3 constructs and maintained for an additional 72 h under standard conditions. Cells were then pre-fixed in 0.4% paraformaldehyde (PFA) in DPBS for 10 min at 37 °C, followed by a 10-min fixation in 4% PFA in DPBS at room temperature. When cells were grown on coverslips, their nuclei were counterstained with VECTASHIELD antifade mounting medium containing DAPI (H-1200–10, Vector Laboratories), whereas plate-grown cells were only overlaid with DPBS. All samples were stored in the dark at 4 °C until imaging. For quantitative analysis, 6 randomly chosen fields of vision per well were captured at 100 × magnification on an Axioplan 2 imaging microscope equipped with an AxioCam MRm camera and AxioVision 4.3 imaging software (all Carl Zeiss, Jena, Germany). Cell and aggregate counts were obtained by manual counting.

### Gene Expression Omnibus

Gene Expression Omnibus (GEO) datasets were downloaded from NCBI, specifically GSE28715, GSE71817, GSE56937, and GSE270493. The downloaded data were normalized and subsequently used for correlation analysis between TFs and *KPNA3*. Correlation analysis was conducted using Pearson correlation coefficients, and scatter plots were generated with GraphPad Prism 10.1.1 (Dotmatics, Boston, MA, USA) to visualize gene expression relationships.

### Human Protein Atlas

The expression levels of various TFs across different regions of the human brain were obtained from Human Protein Atlas (https://www.proteinatlas.org/).

### Statistical analysis

Statistical analyses evaluating the correlation between *KPNA3* SNP rs11618521 and the age at onset (AAO) of SCA3 were conducted in JMP (v16.2.0; JMP, Cary, NC, USA). Significance was defined as *p* ≤ 0.05. Hardy–Weinberg equilibrium for SNP rs11618521 was tested with a *χ*^2^ test. Samples lacking reliable genotyping, CAG repeat size, or AAO data were omitted. Differences in expanded CAG repeat length or the AAO among genotypes were assessed with a two-tailed Kruskal–Wallis test followed by Steel–Dwass post hoc comparisons. Because some individuals were related, each family was treated as a single statistical unit by assigning every family member a weight equal to 1/*n*, where n is the number of family members [58]. Multivariate linear regression with the AAO as the dependent variable and both rs11618521 genotype and expanded CAG repeat length as independent variables quantified the polymorphism’s effect beyond the known CAG influence. Additive model (CAG + rs11618521) was contrasted with a CAG-only model using changes in the coefficient of determination (*r*^2^) and effect tests. Least-squares means were used to adjust the AAO to the cohort’s mean expanded-allele CAG length, enabling genotype-specific comparisons. Genotype-specific linear prediction equations (AAO vs. CAG repeats) were derived for refined AAO estimation. Box plots were produced in GraphPad Prism 10.1.1 (Dotmatics, Boston, MA, USA). Functional-assay data were also analyzed in GraphPad Prism 10.1.1. Results are displayed as means ± SEM bar graphs or box plots (median and interquartile range). Statistical testing employed one-sample or unpaired/paired Student’s *t*-tests, or one-way ANOVA with appropriate post hoc corrections; *p* ≤ 0.05 was considered significant. Additional details are provided in the figure legends.

## Results

### SNP rs11618521 in the *KPNA3* promoter modifies the age at onset of SCA3 patients

**Fig. 1:**
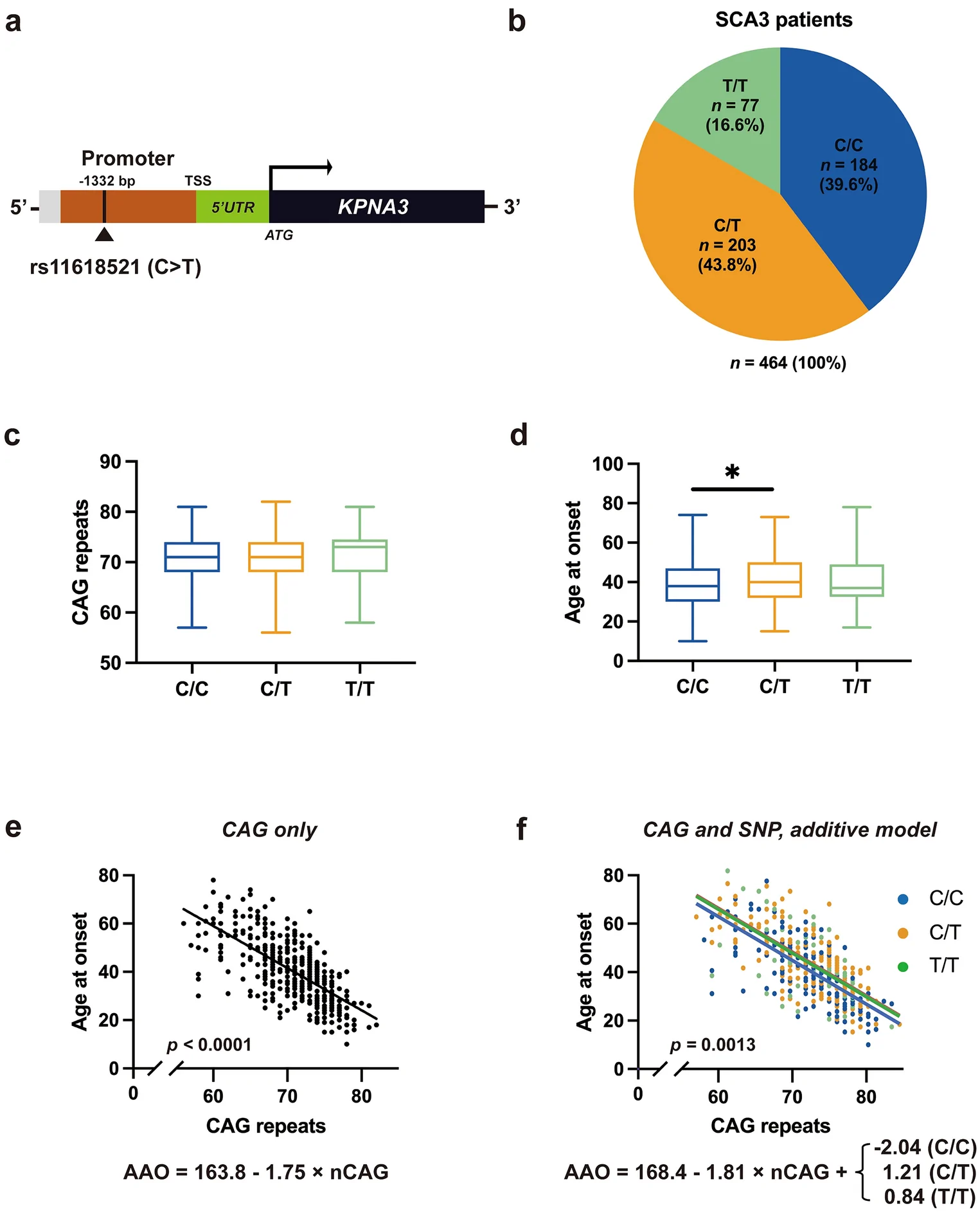
SNP rs11618521 (C > T) in the *KPNA3* promoter region contributes to the age at onset of SCA3. **a** Schematic diagram showing the genomic location of SNP rs11618521 within the *KPNA3* promoter region (marked in brown). SNP rs11618521 is located 1332 bp upstream of the transcription start site (TSS) and 1509 bp upstream of the translation start site (ATG). The *KPNA3* coding region is marked in black and the 5’UTR is marked in green. **b** SNP rs11618521 genotype frequencies in the cohort of SCA3 patients. C is the major allele, while T represents the minor allele with a population frequency of 38.5%. In our cohort, most SCA3 patients (43.8%) were heterozygous for this variant. **c** Box plots showing the distribution of CAG repeat numbers per SNP rs11618521 genotype in SCA3 patients. No statistical difference in the length of CAG repeat number was detected (*p* = 0.60). **d** Box plots showing the distribution of the age at onset (AAO) per SNP rs11618521 genotype in SCA3 patients. A significant genotype effect (*p* = 0.035) was detected. Patients homozygous for the C allele developed symptoms 3.27 years earlier than C/T heterozygotes. Data are presented as means ± SEM; one-way ANOVA followed by multiple comparison tests. **e** Multivariate linear regression analysis between the AAO and the CAG repeat numbers in SCA3 patients. The expected inverse correlation between CAG repeats numbers and the AAO was confirmed (*p* < 0.0001). **f** Additive model of multivariate linear regression analysis between the AAO and SNP rs11618521 genotype in SCA3 patients. Patients homozygous for the major allele C exhibited an approximately 3-year earlier AAO compared with the other genotypes. Notably, the effect of the polymorphism together with the CAG repeats on the AAO reached statistical significance (*p* = 0.0013)

Karyopherin alpha 3 (KPNA3) plays a crucial role in the pathology of SCA3. Therefore, we aimed to identify molecular mechanisms regulating *KPNA3* expression and to assay its impact on the disease. We focused on SNP rs11618521 in the *KPNA3* promoter to investigate its potential association with SCA3, as this variant exhibits a relatively balanced genotype distribution in the population, ensuring adequate sample sizes across genotype groups. Specifically, SNP rs11618521 lies 1332 bp upstream of the *KPNA3* transcription start site and 1509 bp upstream of the first ATG (Fig. 1a). To assess whether SNP rs11618521 contributes to the age at onset (AAO) in SCA3 patients, we first established a high-resolution melting assay capable of distinguishing all three SNP states in DNA samples and genotyped our cohort of SCA3 patients (*n* = 464) (Supplementary Fig. S1a–b). The genotyping results were further validated by Sanger sequencing (Supplementary Fig. S1c). The observed allele distribution (Fig. 1b) was highly consistent with the ALFA reference dataset (Release version: 20250407153717; C, reference allele = 62.7%, T, alternate allele = 37.3%), supporting the reliability of our analysis.

We next evaluated whether the length of the expanded CAG repeat differed among the patients carrying the three SNP rs11618521 genotypes, as such variation could confound potential effects on the AAO of SCA3 patients. No statistically significant differences in mean repeat length were detected (Fig. 1c, Table 1). By contrast, the mean AAO differed markedly by genotype: patients homozygous for the C allele developed symptoms 3.27 years earlier than C/T heterozygotes (*p* = 0.022) (Fig. 1d, Table 1). As the length of the expanded CAG repeat is the principal determinant of the AAO in SCA3 patients, we adjusted the mean AAO for CAG length. After adjustment, patients with the C/C genotype had a mean AAO approximately 3.2 years earlier than those with the C/T genotype (Table 1). In a multivariate linear model, the CAG repeat length proved to be strongly and inversely associated with the AAO, consistent with its dominant effect (*p* < 0.0001) (Fig. 1e, Table 2). We next evaluated whether SNP rs11618521 exerts an additive effect on the AAO in addition to the CAG repeat length. To this end, we fitted an additive linear regression model with the AAO as dependent variable, and the CAG repeat length and SNP rs11618521 genotype as independent variables. The linear equations, including the regression coefficients for each haplotype, are provided at the bottom of the graphs. This model revealed a strong effect of the SNP on the AAO (*p* = 0.0013), suggesting that SNP rs11618521 may additively modify the disease onset (Fig. 1f, Table 2). Notably, more than 60% of SCA3 patients in our cohort were either homozygous or heterozygous carriers of the alternate T allele. We then combined C/T and T/T genotypes for comparison with C/C (Supplementary Fig. S2a). The mean CAG repeat length did not differ between these groups (*p* = 0.91) (Supplementary Fig. S2b), whereas the mean AAO remained significantly different (*p* = 0.01) (Supplementary Fig. S2c). Further multivariate analysis yielded consistent results: C/C carriers exhibited an earlier disease onset by approximately 3 years compared with carriers of at least one T allele (Supplementary Fig. S2d). These findings indicate that the presence of a T allele is sufficient to delay the onset of SCA3. Taken together, we confirmed that the SNP rs11618521 located in the *KPNA3* promoter region genetically modifies the AAO of SCA3 patients.

**Table 2:** Multivariate linear regression analysis with an additive model shows a significant contribution of the *KPNA3* SNP rs11618521 together with the *ATXN3* CAG repeats to the age at onset in the SCA3 cohort

CAG repeats		CAG repeats and SNP, additive model		
*r*^2^	*p* (CAG)	*r*^2^	Δ*r*^2^	*p* (SNP)
0.462	< 0.0001	0.475	0.013	0.0013

Including SNP together with the length of the expanded CAG repeat in the statistical analysis improves the prediction of the age at onset by 1.3 percentage points.* r*^2^: coefficient of determination

### SNP rs11618521 modulates the *KPNA3* promoter activity

Since SNP rs11618521 influences the AAO in SCA3 patients and is located within the *KPNA3* promoter region, where it may regulate *KPNA3* expression, we next sought to determine whether this SNP affects promoter activity and thereby alters *KPNA3* expression.

**Fig. 2:**
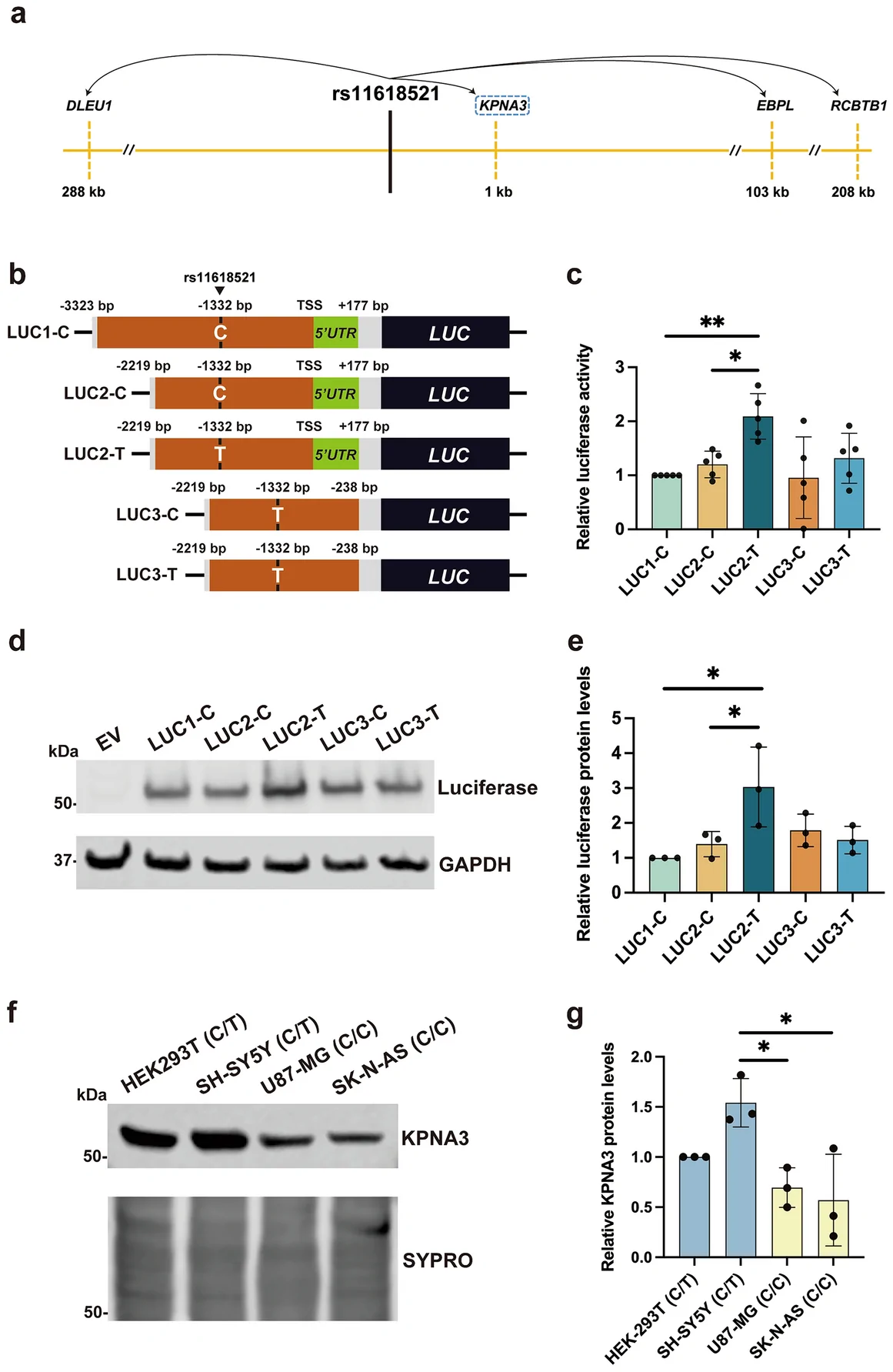
HEK293T cell-based reporter assays reveal that SNP rs11618521 alters *KPNA3* promoter activity. **a** Schematic diagram illustrating SNP rs11618521 and its associated genes whose expression levels are potentially affected by the genetic variants, according to expression quantitative trait loci (eQTL) analysis. The nearest gene is *KPNA3* (approximately 1 kb), with all other genes located at least 100 kb away from SNP rs11618521. *DLEU1*, deleted in lymphocytic leukemia 1; *EBPL*, Emopamil-binding protein like; *RCBTB1*, RCC1 and BTB domain containing protein 1. **b** Reporter constructs containing up to 3323 bp upstream of the transcription start site (TSS) for the dual-luciferase assay. Construct LUC1 contains 3323 bp of sequence upstream of the TSS together with the 5′UTR sequence. Construct LUC2 harbors a shorter upstream sequence (2219 bp) along with the 5′UTR. LUC2-C and LUC2-T differ only in the allelic state of SNP rs11618521 (C or T). Construct LUC3 extends from − 2219 bp to − 238 bp upstream of the TSS. **c** Luciferase activity of the constructs shown in **a** was measured in HEK293T cells following transient transfection. For each experimental condition, firefly luciferase activity was normalized to *Renilla* luciferase activity to account for transfection efficiency and expressed relative to LUC1-C. LUC2-T, which carries the T allele at SNP rs11618521 and includes the 5′UTR, showed significantly higher luciferase activity than LUC2-C carrying the C allele of the SNP. No other constructs showed statistical differences in luciferase activity. Data are presented as means ± SEM. Statistical analysis was performed using one-way ANOVA followed by multiple comparison tests (*p < 0.05, *n* = 5*)*. **d** Western blot analysis of luciferase expression in HEK293T cells after transfection with the respective constructs. As control, cells were transfected with the empty luciferase vector (EV). Luciferase protein expression was higher in cells transfected with LUC2-T compared with control LUC1-C or LUC2-C, consistent with our reporter gene assays. GAPDH served as loading control. *EV* empty vector. **e** Quantification of luciferase protein levels from **d**, normalized to GAPDH. Data are presented as means ± SEM; one-way ANOVA followed by multiple comparison tests (*p < 0.05, *n* = 3*)*. **f** Western blot analysis of KPNA3 protein levels of HEK293T, SH-SY5Y, U87-MG and SK-N-AS cell lines. KPNA3 protein levels were higher in cell lines heterozygous (C/T) for rs11618521 (HEK293T and SH-SY5Y) compared to homozygous (C/C) lines (U87-MG and SK-N-AS). SYPRO was used as the loading control to avoid potential biases introduced by cell type-associated effects on housekeeping protein levels [2, 4]. **g** Quantification of KPNA3 protein levels from **f**, normalized to SYPRO and the HEK293T group. Data are presented as means ± SEM; one-way ANOVA followed by multiple comparison tests (*p < 0.05, *n* = 3*)*

We first examined which genes are affected by SNP rs11618521 using the expression quantitative trait loci eQTLGen consortium database, which investigates the associations between genetic variants and gene expression levels [57]. As expected, the analysis revealed that SNP rs11618521 is associated with altered expression of *KPNA3*, but also with three other genes (*DLEU1*, *EBPL*, *RCBTB1*) which are located at least 100 kb away from SNP rs11618521 (Fig. 2a). The other three genes were excluded from our study owing to the lack of evidence linking them to polyQ-related neurodegenerative diseases and their substantial genomic distance from SNP rs11618521. To further verify whether SNP rs11618521 modulates *KPNA3* expression, we generated luciferase reporter plasmids and evaluated the effect of this SNP on *KPNA3* expression. These constructs were generated for both versions (C or T) of rs11618521. Given that promoter regions lie upstream of genes yet lack sharply defined boundaries, we used an extended ~ 3 kb upstream *KPNA3* sequence as a reference to ensure comprehensive coverage of putative regulatory elements (LUC1-C) as well as constructs containing approximately 2 kb upstream sequence with (LUC2) or without (LUC3) the 5’UTR (Fig. 2b). These constructs were transiently transfected into HEK293T cells which are commonly used as a transfection model system, and promoter activity was assessed by measuring firefly luciferase activity normalized to *Renilla* luciferase as an internal control for transfection efficiency. Our results revealed allele-specific effects on *KPNA3* promoter activity. No significant differences were observed among constructs carrying the C version of the SNP (LUC1-C, LUC2-C and LUC3-C) or lacking the 5’UTR (LUC3-C and LUC3-T). However, the construct containing the T allele together with the 5’UTR (LUC2-T) showed significantly higher promoter activity compared to the same construct containing the C allele (LUC2-C) (*p* = 0.039) (Fig. 2c), which also indicates that the 2.2 kb upstream of the transcription start site is sufficient and that the 5’UTR is required for the SNP’s effect. To further validate this difference in promoter activity, we examined luciferase protein levels in cells transfected with the respective constructs. Consistent with the dual-luciferase assay, western blot analysis confirmed that intracellular luciferase protein levels were significantly higher in cells transfected with LUC2-T compared with the C allele construct (LUC2-C) (*p* = 0.047) (Fig. 2d–e).

After confirming that SNP rs11618521 can affect *KPNA3* promoter activity in our luciferase assay, specifically that the promoter activity of the C allele is lower than that of the T allele—the effect of endogenous rs11618521 on KPNA3 levels required further investigation. Therefore, we identified two cell lines carrying the heterozygous C/T genotype of rs11618521 (HEK293T and SH-SY5Y) and two cell lines carrying the homozygous C/C genotype of rs11618521 (U87-MG and SK-N-AS). Given that the presence of a single T allele is sufficient to exert a significant effect in patients, recruiting a cell line with the rarer T/T genotype was abandoned. By examining endogenous KPNA3 protein levels across cell lines with different genotypes, we found that KPNA3 protein levels were higher in cells carrying the rs11618521 C/T genotype than in those with the C/C genotype (SH-SY5Y vs. U87-MG, *p* = 0.024; SH-SY5Y vs. SK-N-AS, *p* = 0.011) (Fig. 2f–g). This finding is consistent with our previous luciferase reporter results. Although cell type-specific factors cannot be fully excluded, the concordant association between genotype and protein level, together with our prior data, supports that SNP rs11618521 modulates *KPNA3* promoter activity and consequently influences its protein level.

### SNP rs11618521 alters transcription factor-mediated expression of *KPNA3*

SNPs located at promoters frequently modulate transcription factor (TF) binding and, consequently, downstream gene expression [3]. To identify TFs that might differentially recognize SNP rs11618521, we made use of several in silico prediction resources and experimentally derived ChIP-seq datasets. Comparative motif analysis predicted CEBPB, CEBPA, FOXP3, SOX5, SOX10, SOX17, TFAP2A, SOX2 and NANOG as putative allele-selective binders at the *KPNA3* promoter harboring rs11618521, whereas GATA1, PPARG, E2F1 and CTCF were identified based on evidence from public ChIP-seq databases (Supplementary Table S3).

**Fig. 3:**
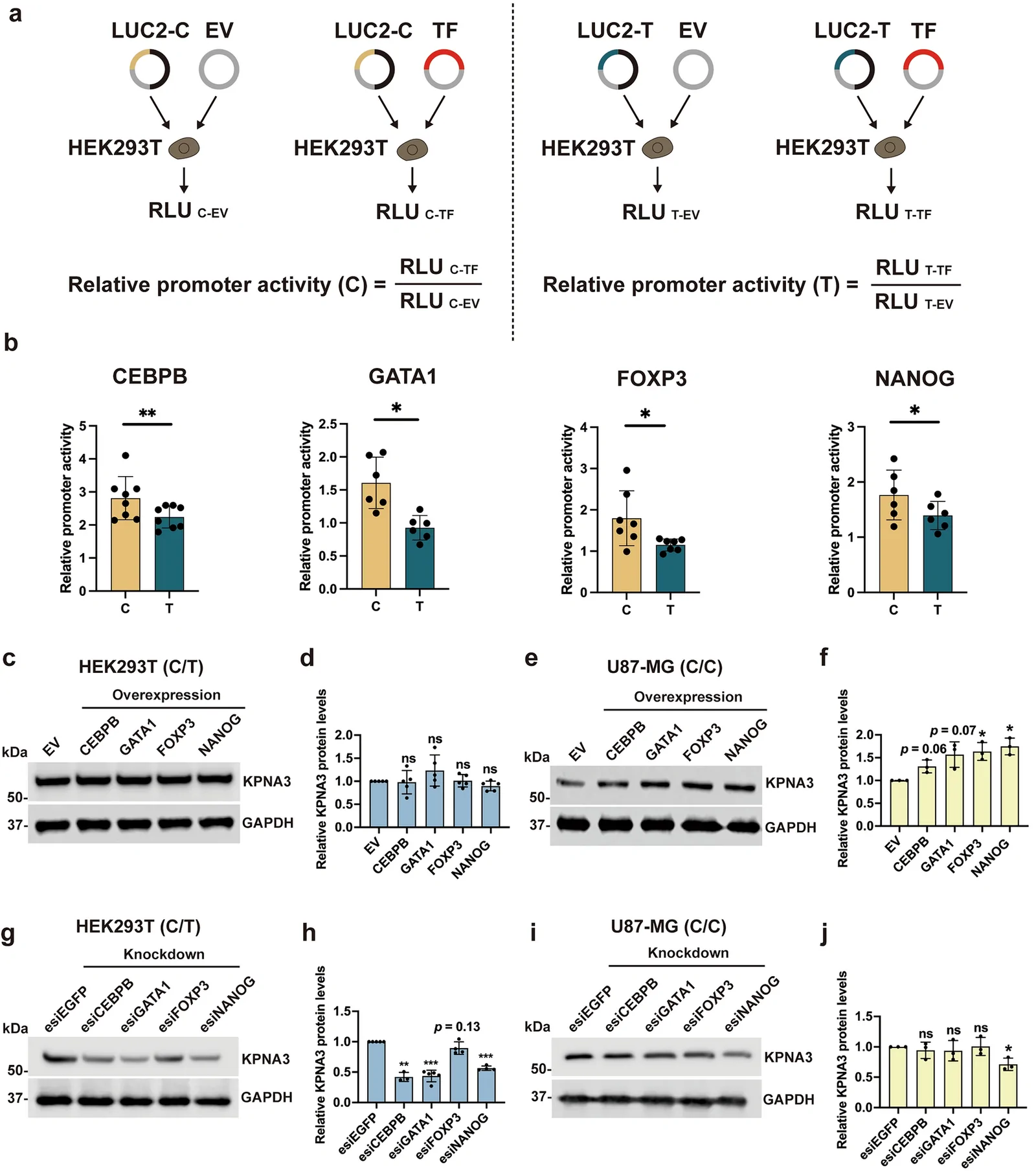
SNP rs11618521 alters transcription factor-mediated regulation of KPNA3 expression and levels. **a** Schematic diagram illustrating the workflow of the dual-luciferase assay evaluating transcription factor (TF)-specific effects at promoters with SNP rs11618521. HEK293T cells were co-transfected with LUC2-C or LUC2-T reporter constructs together with either TF plasmids or empty vectors (EV) and a *Renilla* vector to control the transfection efficiency (not shown here). The relative light units (RLU) were measured and processed to represent the relative promoter activity. **b** Relative activities of the promoters harboring SNP rs11618521 upon co-transfection with screened TFs. CEBPB, GATA1, FOXP3 and NANOG induced a higher promoter activity from the C allele construct than from the T allele construct. Data are presented as means ± SEM; paired *t*-test (*p < 0.05, **p < 0.01, *n* = 6–8). **c** and **e** Western blot analysis of KPNA3 protein levels following TFs overexpression in HEK293T and U87-MG cell lines with different SNP rs11618521 genotypes. Cells were transfected with the empty pcDNA3 vector (EV) as control. In HEK293T (C/T) cells, TFs overexpression did not affect KPNA3 protein levels. By contrast, in U87-MG (C/C) cells, TFs overexpression increased KPNA3 protein levels. GAPDH served as loading control. EV, empty vector. **d** and **f** Quantification of KPNA3 protein levels from **c** and **e**. While not reaching statistical significance, CEBPB and GATA1 overexpression showed trends toward increased KPNA3 levels. Signals normalized to GAPDH. Data are presented as means ± SEM; one-sample *t* and Wilcoxon test (*p < 0.05, *n* = 3–5). **g** and **i** Western blot analysis of KPNA3 protein levels following TFs knockdown by esiRNAs in HEK293T (C/T) and U87-MG (C/C) cell lines. Cells were transfected with esiRNA directed against enhanced green fluorescent protein (esiEGFP) as control. Knockdown of each TF reduced KPNA3 protein levels in HEK293T (C/T) cells. In U87-MG (C/C) cells, only NANOG knockdown resulted in decreased KPNA3 protein levels. GAPDH served as loading control. **h** and **j** Quantification of KPNA3 protein levels from **g** and **i**, normalized to GAPDH. Data are presented as means ± SEM; one-sample* t* and Wilcoxon test (*p < 0.05, *n* = 3–5)

To assess whether individual TFs differentially modulate the activity of the *KPNA3* promoter harboring SNP rs11618521, we established a dual-luciferase, dual-plasmid co-transfection system to quantify relative promoter activity. Briefly, overexpression constructs for candidate TFs were co-transfected with promoter plasmids harboring either allele of rs11618521 (LUC2-C or LUC2-T) together with a *Renilla* (pRL-CMV) control into HEK293T cells. The relative promoter activity driven by each TF in reporter constructs harboring SNP rs11618521 was quantified and normalized to the corresponding control without the TF (Fig. 3a). Our initial screening identified CEBPB, GATA1, FOXP3 and NANOG as TFs exhibiting allele-specific effects at SNP rs11618521 (Supplementary Fig. S3) and was further validated (Fig. 3b), with all showing increased promoter activity with the C allele (CEBPB, *p* = 0.007; GATA1, *p* = 0.023; FOXP3, *p* = 0.018; NANOG, *p* = 0.010). No such effect was observed for other TFs.

To validate our results of the dual-luciferase assays in a physiological genomic context, we next evaluated the effects of these TFs on the endogenous *KPNA3* promoter harboring different SNP rs11618521 alleles. For this, we used HEK293T and SH-SY5Y cell lines representing the C/T genotype and U87-MG and SK-N-AS cell lines for the C/C genotype as genotype-specific models to investigate how promoter variants may alter transcription factor (TF) activity. To gain preliminary insight into potential allele-specific regulation of *KPNA3* by TFs, we first analyzed the correlation between TF and *KPNA3* expression across these cell lines using available RNA-seq data. We retrieved data from four independent RNA-seq experiments involving HEK293T (GEO ID: GSE28715) [52], SH-SY5Y (GEO ID: GSE71817) [30], U87-MG (GEO ID: GSE56937) [35] and SK-N-AS (GEO ID: GSE270493) [39] cell lines. No comparable relationship between the TFs and *KPNA3* was observed in HEK293T or SH-SY5Y cells with the C/T genotype. Notably, in U87-MG and SK-N-AS cells with the C/C genotype, *NANOG* expression is strongly correlated with *KPNA3* (Supplementary Fig. S4).

We then examined the effect of TF overexpressing on KPNA3 protein levels across the four cell lines. Transfection efficiency was first verified by monitoring protein levels of the overexpressed TFs (Supplementary Fig. S5). Subsequent analysis revealed that in HEK293T (C/T) and SH-SY5Y (C/T) cells, KPNA3 protein levels remained unchanged upon TF transfection (Fig. 3c, d, Supplementary Fig. S6A–B). Interestingly, in U87-MG (C/C) cells transfected with FOXP3 or NANOG, KPNA3 protein levels increased (FOXP3 overexpression vs. Ctrl, *p* = 0.03; NANOG overexpression vs. Ctrl, *p* = 0.02), while overexpression of CEBPB and GATA1 showed strong trends in the same direction (CEBPB overexpression vs. Ctrl, *p* = 0.06; GATA1 overexpression vs. Ctrl, *p* = 0.07) (Fig. 3e, f). Similar results for FOXP3 and NANOG overexpression were observed in SK-N-AS (C/C) cells (FOXP3 overexpression vs. Ctrl, *p* = 0.047; NANOG overexpression vs. Ctrl, *p* = 0.048) (Supplementary Fig. S6C–D). These findings are consistent with our earlier observations that the TFs preferentially act on the C allele, suggesting that homozygosity enables more effective activation of the *KPNA3* promoter. Moreover, as we used different cell types, we confirmed that this SNP-specific regulatory relationship is independent of cell type.

For further validation of our overexpression experiments and after confirming that all TFs are expressed across cell lines (Supplementary Fig. S7a), we next performed TF knockdown experiments using esiRNAs. One representative cell line was selected per genotype (HEK293T as C/T and U87-MG as C/C). Knockdown of the respective TF was verified by western blot analysis (Supplementary Fig. S7b–c). In HEK293T (C/T) cells, knockdown of CEBPB, GATA1 and NANOG reduced KPNA3 protein levels (CEBPB knockdown vs. Ctrl, *p* = 0.0054; GATA1 knockdown vs. Ctrl, *p* = 0.0002; NANOG knockdown vs. Ctrl, *p* = 0.0002). Knockdown of FOXP3 also reduced KPNA3 protein levels without reaching statistical significance (*p* = 0.13) (Fig. 3g–h). Notably, in U87-MG (C/C) cells, only NANOG knockdown reduced KPNA3 protein levels (*p* = 0.04) (Fig. 3i–j). These results indicate that, in the heterozygous C/T state, all four TFs can act on the *KPNA3* promoter, whereas in the homozygous C/C state, only NANOG retains promoter engagement. This is consistent with our gene expression-based correlation analysis, in which only *NANOG* correlated with *KPNA3* expression in homozygous cells. These data demonstrate that SNP rs11618521 modulates TF effects at the *KPNA3* promoter and that NANOG exhibits a rs11618521-specific effect on KPNA3 regulation.

To obtain additional evidence supporting NANOG as the lead candidate, we queried the expression profiles of each TF across multiple regions of the human brain using the Human Protein Atlas. Importantly, compared with all other brain regions, *NANOG* expression was markedly enriched in the cerebellum, which is the primary site of SCA3 pathology (Supplementary Fig. S8a) [50, 54]. As NANOG functions as an essential TF maintaining stem cell pluripotency [40] and to further explore the potential relevance of NANOG beyond embryonic contexts, we examined its protein levels across different brain regions (striatum, cortex, cerebellum, hypothalamus, hippocampus, and brainstem) in adult mice (Supplementary Fig. S8b). Consistent with the human data, NANOG exhibited the highest levels in the cerebellum, and this region-specific enrichment was independent of age (Supplementary Fig. S8c). In addition, ChIP-PCR followed by agarose gel electrophoresis was utilized to detect the interaction between NANOG and the *KPNA3* promoter region containing rs11618521. The agarose gel electrophoresis results showed a stronger detection for the input and NANOG than IgG (Supplementary Fig. S9) verifying the interaction of NANOG with the *KPNA3* promoter. Taken together, we selected NANOG (Nanog homeobox; Gene ID 79923) as the focus of our subsequent investigations.

### NANOG modulates ataxin-3 aggregate formation dependent on the* KPNA3* SNP rs11618521

**Fig. 4:**
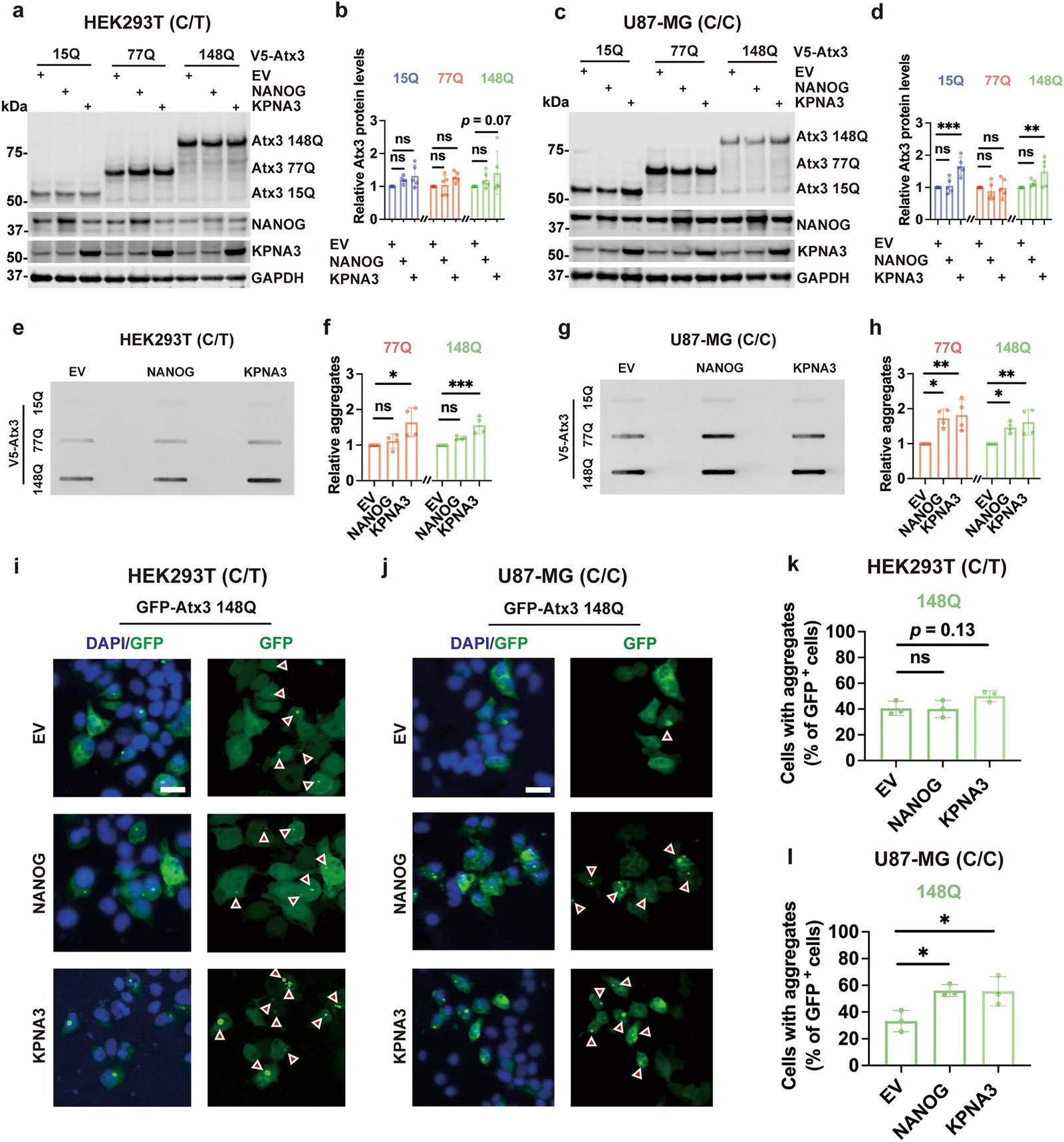
NANOG overexpression differentially modulates aggregate formation in cells with distinct *KPNA3* SNP rs11618521 genotypes. **a** and **c** Western blot analysis of ataxin-3 (Atx3) protein levels following overexpression of NANOG and KPNA3 in HEK293T (C/T) and U87-MG (C/C) cells transfected with V5-Atx3 (15Q, 77Q, 148Q). Membranes were probed with antibodies against the respective protein. KPNA3 overexpression increased soluble ataxin-3 levels in both cell lines overexpressing 148Q. GAPDH served as loading control. EV, empty vector. **b** and **d** Quantification of soluble ataxin-3 levels from **a** and **c**, normalized to GAPDH. Data are presented as means ± SEM; one-sample *t* and Wilcoxon test (**p < 0.01, ***p < 0.001, *n* = 5). **e** and **g** Filter retardation analysis of SDS-insoluble ataxin-3 levels in HEK293T (C/T) and U87-MG (C/C) cells overexpressing NANOG or KPNA3 along with V5-Atx3 15Q, 77Q and 148Q. Interestingly, in U87-MG (C/C) cells, NANOG overexpression mirrored the effect of KPNA3 overexpression, markedly increasing insoluble levels of ataxin-3 77Q and 148Q. In HEK293T (C/T) cells, only KPNA3 overexpression increased levels of insoluble ataxin-3 SDS-insoluble protein species as detected using an anti-ataxin-3 antibody. EV, empty vector.** f** and **h** Quantification of insoluble ataxin-3 levels from **e** and **g**. Data are presented as means ± SEM; one-sample* t* and Wilcoxon test (*p < 0.05, **p < 0.01, ***p < 0.001, *n* = 4). **i** and **j** Fluorescence microscopy showing ataxin-3 aggregates (highlighted by arrowheads) in cells overexpressing NANOG or KPNA3 along with GFP-Atx3 148Q. Aggregate frequency was calculated as the percentage of GFP^+^ cells containing ataxin-3 aggregates relative to the total number of GFP^+^ cells. An increased number of aggregates was present under KPNA3 overexpression in both cell types. Consistent with the filter retardation results, NANOG overexpression led to increased aggregate formation only in U87-MG (C/C) cells only, while showing no effect on aggregate levels in HEK293T (C/T) cells. Scale bar, 20 μm. EV, empty vector. **k** and **l** Quantification of ataxin-3 aggregates from **i** and **j**. Data show the percentage of aggregate-positive cells in GFP^+^ cells. Data are presented as means ± SEM; one-sample *t* and Wilcoxon test (*p < 0.05, *n* = 3)

We next sought to assess the functional role of NANOG in the molecular pathogenesis of SCA3, as this could provide a mechanistic explanation for the effect of SNP rs11618521 in *KPNA3* on the AAO of SCA3 patients. For this, we further employed HEK293T (C/T) and U87-MG (C/C) as representative cell lines of their respective rs11618521 genotypes and overexpressed NANOG or KPNA3 in cells along with ataxin-3 with different polyQ lengths (Atx3 15Q—representing the wild-type protein, Atx3 77Q or 148Q—representing the polyQ-expanded, disease-causative protein). In U87-MG (C/C) cells expressing ataxin-3 15Q or 148Q, overexpression of KPNA3 significantly increased the levels of soluble ataxin-3. By contrast, overexpression of NANOG did not affect soluble ataxin-3 levels in either HEK293T or U87-MG cells (Fig. 4a–d). Aligning with our previous observation, NANOG overexpression elevates KPNA3 levels in U87-MG (C/C) cells (Supplementary Fig. S10). However, insoluble levels of ataxin-3 77Q and 148Q were increased by both NANOG (77Q *p* = 0.013, 148Q *p* = 0.035) and KPNA3 (77Q *p* = 0.007, 148Q *p* = 0.008) overexpression in U87-MG (C/C) cells, while only KPNA3 overexpression triggered the same response in HEK293T (C/T) cells (77Q *p* = 0.013, 148Q* p* = 0.006) (Fig. 4e–h). Supporting this, fluorescence microscopy of GFP-tagged ataxin-3 148Q-expressing cells confirmed that NANOG overexpression enhanced aggregate levels only in U87-MG (C/C) cells (*p* = 0.026), mirroring the effect of KPNA3 (Fig. 4i–l). Collectively, these results support a NANOG–KPNA3 axis that promotes accumulation of polyQ-expanded ataxin-3 aggregates in a SNP rs11618521-specific genetic background.

### Pharmacological inhibition of NANOG reduces KPNA3 protein levels and ataxin-3 aggregates

Given that NANOG regulates KPNA3 protein levels in a SNP rs11618521-specific manner, we next employed Amcasertib (BBI-503), a NANOG inhibitor [21], to explore the therapeutic potential of pharmacologically modulating NANOG in SCA3. Previous research and clinical applications of this compound in tumor therapy support its potential for translation to neurodegenerative diseases [21]. We first optimized the working concentration of Amcasertib by assessing cell viability across our multiple cell lines. Treatment with 0.5 µM or 1 µM Amcasertib for 48 h did not impair cell viability in any line, and these concentrations were therefore selected for subsequent experiments (Supplementary Fig. S11a).

**Fig. 5:**
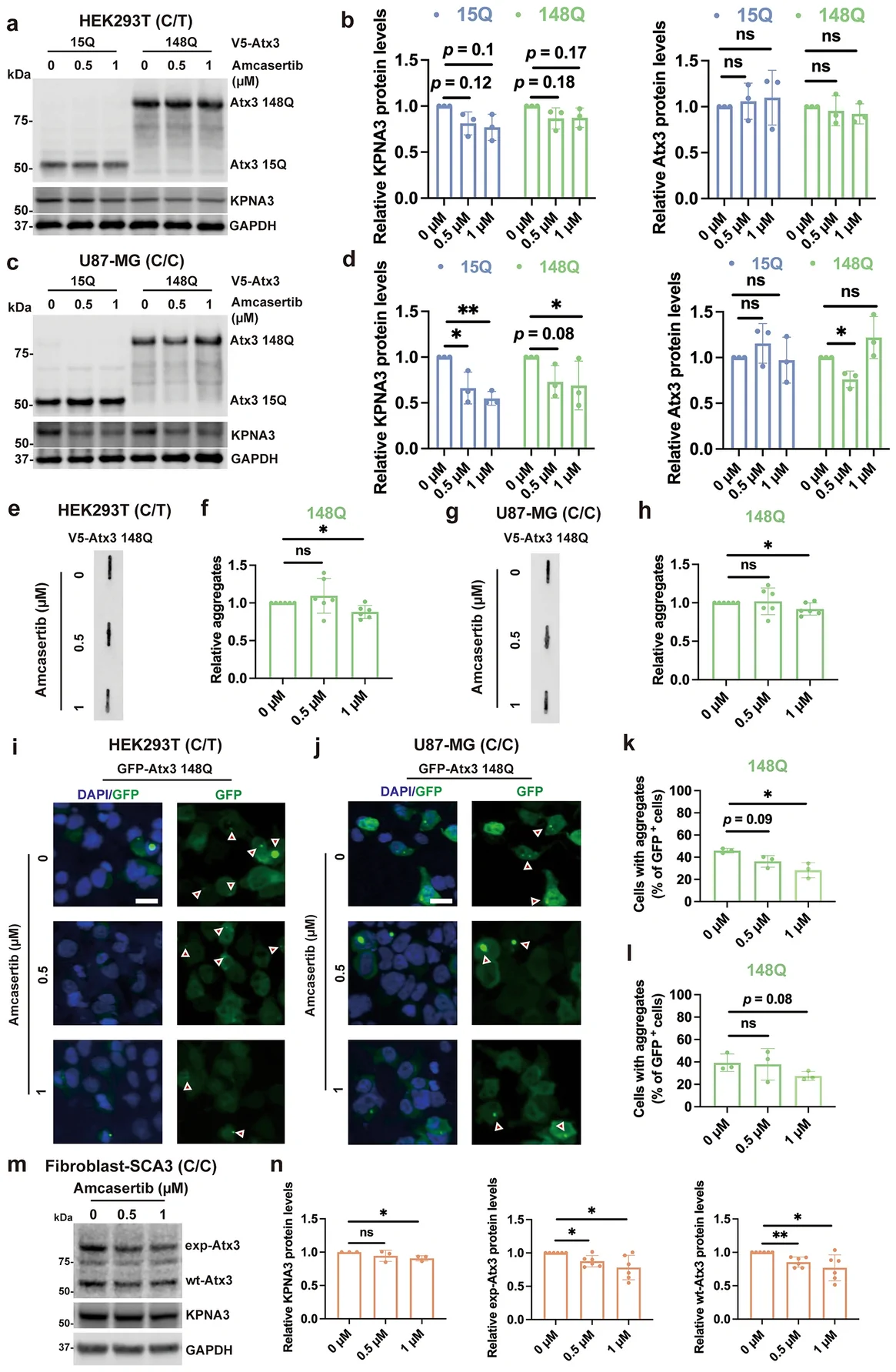
Effect of pharmacological NANOG inhibition on soluble and aggregated ataxin-3. **a** and **c** Western blot analysis of ataxin-3 and KPNA3 protein levels in HEK293T (C/T) and U87-MG (C/C) cells expressing V5-Atx3 15Q or 148Q and treated with 0, 0.5, or 1 µM Amcasertib, an inhibitor of NANOG for 48 h. Membranes were probed with antibodies against the respective protein. GAPDH served as loading control. **b** and **d** Quantification of ataxin-3 and KPNA3 protein levels from **a** and **c**, normalized to GAPDH. Amcasertib treatment reduced KPNA3 protein levels in U87-MG (C/C) cells, whereas a trend towards a level lowering was observed in HEK293T (C/T) cells without reaching statistical significance. A reduction of ataxin-3 protein level in U87-MG (C/C) cells following treatment with 0.5 µM Amcasertib was observed. Data are presented as means ± SEM; one-sample *t* and Wilcoxon test (*p < 0.05, **p < 0.01, *n* = 3). **e** and **g** Filter retardation analysis of SDS-insoluble ataxin-3 levels in HEK293T (C/T) and U87-MG (C/C) cells expressing V5-Atx3 148Q and treated with 0, 0.5 or 1 µM Amcasertib for 48 h. The levels of SDS-insoluble ataxin-3 decreased in both cell lines following treatment with 1 µM Amcasertib. SDS-insoluble protein species were detected using an ataxin-3-specific antibody. **f** and **h** Quantification of ataxin-3 aggregate levels from **e** and **g**. Data are presented as means ± SEM; one-sample *t* and Wilcoxon test (*p < 0.05, *n* = 6). **i** and **j** Fluorescence microscopy showing ataxin-3 aggregates (highlighted by arrowheads) in HEK293T (C/T) and U87-MG (C/C) cells expressing GFP-Atx3 148Q and treated with 0, 0.5, or 1 µM Amcasertib for 48 h. Aggregate frequency was calculated as the percentage of GFP^+^ cells containing ataxin-3 aggregates relative to the total number of GFP^+^ cells. A reduction in ataxin-3 aggregates was observed under 1 µM Amcasertib treatment in both cell types expressing GFP-Atx3 148Q. Scale bar, 20 μm. **k** and **l** Quantification of ataxin-3 aggregates from **i** and **j**. Data show the percentage of aggregate-positive cells in GFP^+^ cells. Data are presented as means ± SEM; one-sample *t* and Wilcoxon test (*p < 0.05, **p < 0.01, *n* = 3). **m** Western blot analysis of expanded and wild-type ataxin-3 and KPNA3 protein levels in fibroblasts derived from an SCA3 patient (C/C) under Amcasertib treatment. Amcasertib treatment reduced both expanded and wild-type ataxin-3, as well as KPNA3 protein levels in SCA3 fibroblasts. GAPDH served as loading control. **n** Quantification of ataxin-3 and KPNA3 levels from **m**, normalized to GAPDH. Data are presented as means ± SEM; one-sample *t* and Wilcoxon test (*p < 0.05, *n* for KPNA3 detection = 3, *n* for ataxin-3 detection = 6)

To evaluate the effects of Amcasertib on ataxin-3, HEK293T (C/T) and U87-MG (C/C) cells were transfected with ataxin-3-expressing constructs with 15Q or 148Q and treated with Amcasertib for 48 h. Western blot analysis showed reduction of KPNA3 protein levels in U87-MG (C/C) cells upon Amcasertib treatment, while a similar but not significant effect was observed in HEK293T (C/T) cells. Notably, 0.5 µM Amcasertib decreased soluble 148Q ataxin-3 in U87-MG (C/C) cells (*p* = 0.045) (Fig. 5a–d). To control for cell type-specific effects, we further examined SH-SY5Y (C/T) and SK-N-AS (C/C) cells. Consistent with the genotype dependence, Amcasertib reduced KPNA3 levels in SK-N-AS (C/C) (1 µM, *p* = 0.020) but not in SH-SY5Y (C/T) cells.

We next examined the effect of Amcasertib on a more pathogenic phenotype: ataxin-3 aggregation. Both filter retardation assays and fluorescence microscopy assays demonstrated that treatment with 1 µM Amcasertib markedly reduced 148Q ataxin-3 aggregates in HEK293T (C/T) and U87-MG (C/C) cells (filter retardation: HEK293T, *p* = 0.020; U87-MG, *p* = 0.051; fluorescence microscopy: HEK293T, *p* = 0.047; U87-MG, *p* = 0.060) (Fig. 5e–l). Similarly, aggregate levels were decreased in SK-N-AS (C/C) cells (*p* = 0.018) but remained unchanged in SH-SY5Y (C/T) cells upon Amcasertib treatment (Supplementary Fig. S11b–e). This pattern mirrors our previous findings that Amcasertib failed to suppress KPNA3 protein expression in SH-SY5Y (C/T) cells, supporting the hypothesis that the aggregate-reducing effect of Amcasertib is likely mediated through KPNA3 downregulation (Supplementary Fig. S11f–m).

Building on our previous observations of the pronounced effects of Amcasertib in SCA3 cell models, we sought to obtain additional clinically relevant evidence. To this end, we employed immortalized fibroblasts which were derived from an SCA3 patient as a model to assess the potential effects of Amcasertib. Genotyping of the fibroblasts for SNP rs11618521 revealed a C/C genotype. Using the same Amcasertib treatment, we observed a significant reduction in KPNA3 protein levels at 1 µM (*p* = 0.050). This effect was accompanied by a decrease of both wild-type (0.5 µM, *p* = 0.005; 1 µM, *p* = 0.033) and polyQ-expanded (0.5 µM, *p* = 0.016; 1 µM, *p* = 0.033) ataxin-3 protein levels (Fig. 5m–n). Taken together, these findings identify NANOG as a therapeutic target for SCA3 and suggest that its inhibitor, Amcasertib, may represent a potential therapeutic compound.

## Discussion

Karyopherin α-3 (KPNA3) shuttles polyglutamine (polyQ)-expanded ataxin-3, the affected protein in SCA3, to the nucleus and contributes to the molecular pathogenesis of this neurodegenerative disease [51]. In a large patient cohort, we identified a *KPNA3* promoter variant, rs11618521, as a genetic modifier of the disease age at onset (AAO). Using cell-based assays, we demonstrated that this variant alters *KPNA3* promoter activity. Mechanistically, our data show that the rs11618521 genotype modulates the transcription factor (TF)-dependent *KPNA3* expression, with NANOG emerging as an allele-specific regulator. Genetic or pharmacological modulation of NANOG in SCA3 cell models and patient-derived fibroblasts influenced ataxin-3 levels and aggregation in a rs11618521 genotype-dependent manner, consistent with changes in KPNA3 levels.

Beyond the well-established inverse correlation between the CAG repeat length and the AAO in SCA3 [31], genetic modifiers are thought to explain why patients with similar repeat lengths exhibit a marked variability in the disease onset—a phenomenon observed across polyQ diseases [12]. Among such modifiers, single nucleotide polymorphisms (SNP)—the most common form of human genetic variations—have repeatedly been associated with differences in the AAO in SCA3 [18]. However, only a limited number of these modifiers have been mechanistically characterized, highlighting the need for further studies. Here, we focused on the gene *KPNA3*, which encodes the nuclear transport protein KPNA3. We have previously demonstrated that KPNA3 promotes polyQ-expanded ataxin-3 toxicity by regulating its intranuclear import, accumulation, and aggregation, thereby influencing SCA3 pathology [51]. Because KPNA3 reduction ameliorated disease phenotypes, we sought to determine whether promoter-region SNPs could contribute to the disease. We selected the SNP rs11618521, located upstream of the *KPNA3* transcription start site, and examined its association with the AAO in a large SCA3 cohort. Correlation analyses revealed an additive effect of the CAG repeat length and rs11618521 genotype on the AAO, with individuals homozygous for the major allele (C/C) showing an approximately 3-year earlier disease onset than carriers of the minor allele (T). This effect size is comparable with findings for other SCA3 genetic modifiers, such as rs1801582 in *PRKN* [33, 44, 58]. However, given the complexity of SCA3 pathogenesis and the presence of multiple potential genetic modifiers, it is challenging to fully control or account for all modifier effects in a clinical cohort. Therefore, in-depth functional analyses are needed to define the contribution of rs11618521 to *KPNA3* regulation and SCA3-related cellular phenotypes.

Expression quantitative trait locus (eQTL) analysis revealed that SNP rs11618521 is associated with the expression levels of *KPNA3*, as well as *DLEU1*, *EBPL* and *RCBTB1*. While *DLEU1*, *EBPL*, and *RCBTB1* show little potential for a relevance in neuronal or neurodegenerative functions, KPNA3 has been experimentally validated as a key mediator of SCA3 pathology [51], reinforcing its role as the major effector gene influenced by rs11618521. To investigate the functional impact of rs11618521 on *KPNA3* expression, we used promoter–reporter constructs and detected allele-dependent differences in promoter activity. Because promoter SNPs can alter transcription by changing TF occupancy [29], we screened for TFs differentially binding to rs11618521 and identified CEBPB, GATA1, FOXP3 and NANOG as potential allele-specific regulators. While CEBPB, GATA1, and FOXP3 acted on the T allele, NANOG specifically interacted with the C allele. Promoter variants that alter TF binding are well documented. For example, the risk allele of an *IFITM3* promoter variant reduces the binding of interferon regulatory factors while increasing CTCF occupancy [3]. Similarly, variants linked to prostate cancer affect the binding of TFs such as NKX3-1 and YY1 [23]. The observed allele-specific TF interaction at rs11618521 provides a possible explanation for the differential *KPNA3* promoter activity.

In neurodegenerative disorders, both TFs and regulatory SNPs are known to modulate pathology. In Parkinson’s disease, *SNCA* expression is influenced by TFs such as ZSCAN21 and GATA2 [9, 14, 45], while in Alzheimer’s disease (AD), SMAD4 exhibits allele-specific binding to AD-associated SNPs, affecting the expression of disease linked genes [19]. Similarly, in Huntington’s disease, a promoter SNP in *HTT* alters NF-κB binding, acting as a modifier of gene expression and disease onset [5]. Our data support a comparable mechanism in SCA3, in which rs11618521 modulates KPNA3 expression via NANOG-mediated regulation, thereby influencing disease relevant phenotypes.

NANOG is a core stem cell regulator that maintains embryonic stem cell pluripotency and self-renewal during early development [38] and plays a key role in cancer biology [24]. In the nervous system, NANOG contributes to cerebellar morphogenesis by regulating the balance between self-renewal and differentiation of cerebellar progenitor cells [8]. Moreover, NANOG has been reported to be associated with neuroinflammatory [62] and neurodegenerative [10, 22] processes. These observations prompted us to investigate the potential role of NANOG in SCA3 pathogenesis. Notably, we observed enriched NANOG expression in the adult mouse cerebellum, supporting its possible involvement in disease development. To test NANOG’s role in SCA3 within the context of the *KPNA3* promoter SNP, we analyzed ataxin-3 aggregation—a hallmark of SCA3 [60]—in cellular models differing by the rs11618521 genotype. We employed four cell lines of distinct origins and rs11618521 genotypes as models to investigate the influence of this SNP on TF-mediated regulatory effects. SH-SY5Y (genotype C/T, human neuroblastoma), U87-MG (genotype C/C, human glioblastoma astrocytoma) and SK-N-AS (genotype C/C, human neuroblastoma) are all cell lines of neural origin that have been widely used as in vitro models for studying neurodegenerative diseases [28, 55, 61]. Although HEK293T cells (genotype C/T, human embryonic kidney cells) are not directly derived from neural tissue, they have been reported to exhibit neuronal characteristics [49]. Thus, despite their different origins, these cell lines are suitable and representative to explore the allele-specific regulatory mechanisms associated with rs11618521. We found that NANOG modulates KPNA3 protein levels and consequently ataxin-3 aggregation in a genotype-dependent manner, with the C/C genotype driving KPNA3 levels and consequently aggregation of polyQ-expanded ataxin-3. The direction of this regulation aligns with our prior findings that nuclear localization and KPNA3-mediated transport are critical for ataxin-3 toxicity [51].

TFs are increasingly explored as therapeutic targets in neurodegeneration [27, 32, 43], although direct genetic modulation of their expression is generally infeasible. Given the prominent role of NANOG in our SCA3 models, we examined whether pharmacological inhibition of NANOG could mitigate SCA3-related molecular pathology. We evaluated Amcasertib (BBI503), an oral, first-in-class “stemness” kinase inhibitor, which was previously shown to inhibit NANOG [21, 25]. Early-phase clinical studies, including patients with adenoid cystic carcinoma, have reported acceptable safety and preliminary efficacy [16]. In three of our four SCA3 cellular models, Amcasertib reduced KPNA3 protein levels and decreased ataxin-3 aggregation. Each of these cell lines contains at least one C allele, which is potentially regulated by NANOG. However, no effect was observed in SH-SY5Y cells, possibly reflecting that SH-SY5Y cells exhibit particularly higher activity of ATP-binding cassette efflux transporters (e.g., ABCB1/MDR1) than other used cell lines, which can limit intracellular drug accumulation and reduce apparent uptake [17, 41]. In SCA3 patient-derived fibroblasts, which lack aggregation phenotypes, Amcasertib likewise decreased KPNA3 levels, and notably reduced soluble ataxin-3; although these observations were derived from fibroblasts with a single genotypic background, they suggest additional cell type-dependent regulatory effects that may be related to endogenous ataxin-3 levels, warranting further investigation across a broader range of genotypes. Collectively, these findings identify the NANOG–KPNA3–ataxin-3 axis as a pharmacologically tractable pathway in SCA3.

Despite providing extensive statistical and mechanistic evidence linking the *KPNA3* rs11618521 variant and NANOG to SCA3 pathogenesis, we cannot rule out that our observations may be influenced, at least in part, by cell type-specific factors. Nevertheless, we observed genotype-specific, convergent disease-associated phenotypes across three distinct cell lines, suggesting that the effect is not confined to a single cellular context. To further strengthen these findings, future studies should employ rs11618521-targeted CRISPR-edited isogenic cell lines. In addition, given that ataxin-3 aggregates are present beyond the cerebellum [46], future studies should also consider the potential roles of other TFs.

In summary, we identified a noncoding *KPNA3* promoter variant, rs11618521, that modulates *KPNA3* transcription and thereby contributes to SCA3 molecular pathogenesis, influencing both disease pathomechanism and the AAO. Further characterization of this variant’s functional consequences may improve prognostic precision in SCA3 and reveal broader implications across neurodegenerative diseases. Importantly, our results highlight NANOG as a regulator of neurodegeneration-relevant mechanisms through modulation of *KPNA3*, representing a potential therapeutic target for SCA3 and other conditions involving this nuclear transport pathway.

## Supplementary Information

Below is the link to the electronic supplementary material.

Supplementary file1 (DOCX 50088 KB)

## Data Availability

In our study, we analyzed the data from the previously published datasets GSE288715, GSE71817, GSE56937, and GSE270493. All other data generated in this study are included in the manuscript and supplementary files.
