# Supplementary material for: SNP rs11618521 modifies transcription factor NANOG-mediated expression of KPNA3 and the age at onset of spinocerebellar ataxia type 3
Source: Acta Neuropathol. 2026 Oct 30;152(1):63. doi: 10.1007/s00401-026-03051-0 (PMC13722750; doi:10.1007/s00401-026-03051-0)
Supplement: Supplementary file 1 [file 401_2026_3051_MOESM1_ESM.docx]

**Supplementary file 1**

**SNP rs11618521 Modifies Transcription Factor NANOG Mediated Expression of KPNA3 and the Age at Onset of Spinocerebellar Ataxia Type 3**

Hezhou Han^1^, Leah Czisch^1^, Manuel Pascal Wanner^1^, Jonasz Jeremiasz Weber^1,2^, Miriam Christina Dieterle^1^, Carolina Alexandra Licia Schroeder^1^, Pengfei Qi^1,2^, Junbo Zhou^1^, Jaqueline Jung^1^, Wenxu Zheng^1^, Fubo Cheng^1^, Priscila Pereira Sena^1^, Florian Fath^1,2^, Gunnar Blumenstock^3^, Carlos R Gordon^4^, Roy Zaltzman^4^, Marcondes C França Jr^5^, Maria-Luiza Saraiva-Pereira^6,7,8^, Laura Bannach Jardim^6,8,9^, Mario R Cornejo-Olivas^10,11^, Peter Bauer^1,12,13^, Ludger Schöls^14,15^, Bart P C van de Warrenburg^16^, Alexandra Durr^17^, Alexis Brice^17^, Thomas Klockgether^18^, Olaf Riess^1^, The EUROSCA Network and Thorsten Schmidt^1*^

^1^ Institute of Medical Genetics and Applied Genomics, University of Tübingen, 72076 Tübingen, Germany

^2^ Department of Human Genetics, Ruhr University Bochum, 44801 Bochum, Germany

^3^ Department of Clinical Epidemiology and Applied Biometry, University of Tübingen, 72076 Tübingen, Germany

^4^ Department of Neurology, Meir Medical Center, Tel Aviv University, 69978 Tel Aviv, Israel

^5^ Universidade Estadual de Campinas (UNICAMP), Campinas, 13083-970 Brazil

^6^ Serviço de Genética Médica, Hospital de Clínicas de Porto Alegre, Porto Alegre, 90035-903 Brazil

^7^ Departamento de Bioquímica, Universidade Federal do Rio Grande do Sul, Porto Alegre, 90035-003 Brazil

^8^ Centros de Pesquisa Clínica e Experimental, Hospital de Clinicas de Porto Alegre, 90035-903, Porto Alegre, Brazil

^9^ Departamento de Medicina Interna, Faculdade de Medicina, Universidade Federal do Rio Grande do Sul, Porto Alegre, 90035-903 Brazil

^10^ Neurogenetics Working Group, Universidad Científica del Sur, 15067 Lima, Peru

^11^ Neurogenetics Research Center, Instituto Nacional de Ciencias Neurológicas, 15003 Lima, Peru

^12^ Centogene GmbH, 18055 Rostock, Germany

^13^ Pomeranian Medical University PUM, 70-204, Szczecin, Poland

^14^ Department of Neurology and Hertie-Institute for Clinical Brain Research, University of Tübingen, 72076 Tübingen, Germany

^15^ German Center of Neurodegenerative Diseases (DZNE), 72076 Tübingen, Germany

^16^ Department of Neurology, Donders Institute for Brain, Cognition, and Behaviour, Radboud University Medical Center, 6525 Nijmegen, Netherlands

^17^ Sorbonne Université, Institut du Cerveau – Paris Brain Institute – ICM, Inserm, CNRS, Assistance Publique Hôpitaux de Paris, Hôpital Pitié-Salpêtrière, Paris, France

^18^ German Center for Neurodegenerative Diseases (DZNE), 53127 Bonn, Germany

^*^Correspondence should be addressed to:

Dr. Thorsten Schmidt, PhD, MME, Institute of Medical Genetics and Applied Genomics, University of Tübingen, Nägelestraße 5, 72074 Tübingen, Germany; Phone: +49 7071 29 72277; Fax: +49 7071 29 5228; e-mail: [thorsten.schmidt@med.uni-tuebingen.de](mailto:thorsten.schmidt@med.uni-tuebingen.de)

**Supplementary figures and figure legends**

**Supplementary Fig. S1** Genotyping of SNP rs11618521 by high-resolution melting analysis and Sanger sequencing

**a** Raw fluorescence curves obtained during high-resolution melting (HRM) of amplicons from SCA3 patients encompassing SNP rs11618521. The pre-melt (76.51–78.08 °C) and post-melt (82.39–83.06 °C) slider settings used for normalization in the LightCycler software are indicated below the graph. **b** Normalized and temperature-shifted difference plots clearly distinguish the three genotypes: T/T (green), C/T (blue) and C/C (red). **c** Representative Sanger sequencing chromatograms validating the HRM calls. The polymorphic nucleotide is highlighted by an arrow in each trace, confirming homozygous C/C (top), heterozygous C/T (middle) and homozygous T/T (bottom) genotypes.

**Supplementary Fig. S2** Differences in age at onset between SCA3 patients carrying the SNP rs11618521 genotype C/C and patients carrying either C/T or T/T genotypes

**a** Genotype frequency of C/C versus the combined C/T and T/T in the cohort of SCA3 patients. **b** Box plots showing no statistical difference in the length of CAG repeats between C/C versus the combined C/T and T/T genotypes (*p* = 0.91). **c** Box plots showing the distribution of the age at onset (AAO) in SCA3 patients with the C/C genotype compared with the combined C/T and T/T genotypes. A statistically significant genotype effect was detected (*p* = 0.01), with the AAO being significantly lower in patients carrying the C/C genotype than in those carrying at least one T allele. Data are presented as means ± SEM; unpaired *t*-test. **d** Additive model of multivariate linear regression analysis of AAO in relation to the C/C genotype versus the combined C/T and T/T genotypes. SCA3 patients with the C/C genotype showed an approximately 3-year earlier AAO compared with those carrying at least one T allele (*p* = 0.0003).

**Supplementary Fig. S3** Screening of transcription factors which potentially exert different effects on the *KPNA3* promoter carrying SNP rs11618521 variant C or T

Relative promoter activities of the promoter reporter plasmids LUC2-C and LUC2-T driven by each TF was measured in HEK293T cells. CEBPB, GATA1, FOXP3, and NANOG revealed higher relative promoter activity from the C-allele construct compared with the T-allele construct (CEBPB, *p* = 0.009; GATA1, *p* = 0.003; FOXP3, *p* = 0.049; NANOG, *p* = 0.028). Each bar represents the mean of four technical replicates (individual data points shown). Data are presented as means ± SEM; unpaired *t* test (**p* < 0.05, ***p* < 0.01).

**Supplementary Fig. S4** Correlation between *KPNA3* and transcription factor expression across cell lines with distinct rs11618521 genotypes based on RNA-sequencing data

Normalized RNA-seq data for *CEBPB*, *GATA1*, *FOXP3* and *NANOG* were extracted from four independent GEO datasets (**a** HEK293T from GSE28715, **b** SH-SY5Y from GSE71817, **c** U87-MG from GSE56937, and **d** SK-N-AS from GSE270493) and plotted against *KPNA3* mRNA abundance. Each dot represents one biological replicate, and solid lines depict the best-fit linear regression. Pearson correlation coefficients (r) and two-tailed *p* values are shown in each panel. In the homozygous C/C background (U87-MG and SK-N-AS), *NANOG* exhibited a strong positive correlation with *KPNA3* (U87-MG, *r* = 0.68, *p* < 0.0001; SK-N-AS, *r* = 0.59, *p* = 0.004, *p* values highlighted in red), whereas no significant associations were detected in heterozygous C/T cell lines (HEK293T and SH-SY5Y) or with any other TFs analyzed.

**Supplementary Fig. S5** Validation of transcription factor overexpression across various cell lines

Western blot analysis of transcription factor expression following transfection of respective expression constructs into **a** HEK293T (C/T), **b** SY-SY5Y (C/T), **c** U87-MG (C/C) and **d** SK-N-AS (C/C) cell lines. Cells were transfected with the empty pcDNA3 vector (EV) as control. Membranes were probed with respective antibodies. Since the GATA1 expressing plasmid encodes a 6×His Tag, a His tag-specific antibody was used to detect exogenous GATA1. GAPDH served as loading control. EV, empty vector.

**Supplementary Fig. S6** Impact of transcription factor overexpression on KPNA3 protein levels in SH-SY5Y (C/T) and SK-N-AS (C/C) cells

**a** and **c** Western blot analysis of KPNA3 levels in SH-SY5Y (C/T) and SK-N-AS (C/C) cell lines following overexpression of respective transcription factors (TFs). Cells were transfected with the empty pcDNA3 vector as control. Consistent with the results from the other two cell lines, TF overexpression did not alter KPNA3 protein levels in SH-SY5Y (C/T) cells. In contrast, in SK-N-AS (C/C) cells, overexpression of FOXP3 or NANOG increased KPNA3 protein levels (FOXP3, *p* = 0.047; NANOG, *p* = 0.048). Together with the results from the other two cell lines (HEK293T and U87-MG), these findings indicated that SNP rs11618521 alters the ability of TFs to bind to the promoter. GAPDH served as loading control. EV, empty vector. **b** and **d** Quantification of KPNA3 protein levels from **a** and **c**, normalized to GAPDH. Data are presented as means ± SEM; one-sample *t* and Wilcoxon test (**p* < 0.05, *n* = 3–4); ns, not significant.

**Supplementary Fig. S7** Validation of transcription factor levels and knockdown across various cell lines

**a** Western blot analysis of transcription factor (TF) levels in HEK293T (C/T), SH-SY5Y (C/T), U87-MG (C/C) and SK-N-AS (C/C). TFs showed detectable levels in the examined cell lines, providing a foundation for the subsequent knockdown experiments. **b** and **c** Western blot analysis of TF protein levels in HEK293T (C/T) and U87-MG (C/C) cells following esiRNA-mediated knockdown of the respective TFs. Cells were transfected with esiRNA directed against enhanced green fluorescent protein (esiEGFP) as control. Membranes were probed with antibodies against respective proteins, validating successful lowering of TF levels upon esiRNA-mediated knockdown. GAPDH served as loading control.

**Supplementary Fig. S8** NANOG exhibits region-specific high expression in the cerebellum

**a** Bar graphs showing normalized transcripts per million (nTPM) from the Human Protein Atlas (https://www.proteinatlas.org/) across thirteen macro-dissected regions of the adult human brain. NANOG is expressed at higher levels in the human cerebellum (indicated by an arrowhead), whereas no comparable region-specific enrichment was observed for the other transcription factors. **b** Schematic diagram illustrating the specific regions from which mouse brain samples were collected. The graphical elements in this figure were adapted from illustrations by Luigi Petrucco and Federico Claudi (doi.org/10.5281/zenodo.3925911), available from the SCI-draw platform (https://scidraw.io/). **c** Western blot analysis of NANOG expression in different brain regions of C57BL/6N mice at various ages. NANOG exhibited relatively higher expression in the cerebellum compared with other brain regions across all ages examined. SYPRO served as loading control. **d** Quantification of NANOG expression from **c**, normalized to SYPRO. *n* = 1. Cortex refers to the cerebral cortex in **b**, **c** and **d**.

**Supplementary Fig. S9** ChIP-PCR assay demonstrates that NANOG directly binds to the *KPNA3* promoter region containing rs11618521

**a** Representative agarose gel electrophoresis image of PCR products amplified from chromatin immunoprecipitation (ChIP) samples using anti-NANOG antibody or IgG negative control. Input DNA was used as a positive control. **b** Quantitative ChIP-PCR analysis presented as percentage of input DNA (% of input). Enrichment of the *KPNA3* promoter region containing rs11618521 was significantly increased in the NANOG immunoprecipitation group compared with the IgG control, indicating direct binding of NANOG to the *KPNA3* promoter. Data are presented as mean ± SEM; one-way ANOVA (****p* < 0.001, *n* = 5).

**Supplementary Fig. S10** Quantification of NANOG and KPNA3 protein levels after transfection with the respective expression plasmids

Quantification of NANOG and KPNA3 protein levels from western blot analysis shown in Fig 4 **a** and **c**. Signals were normalized to GAPDH. Results demonstrate the successful overexpression of TF in transfected cells. NANOG overexpression increased KPNA3 protein levels in U87-MG (C/C) cells, but not HEK293T (C/T) cells. Data are presented as means ± SEM; one-sample *t* and Wilcoxon test (**p* < 0.05, ***p* < 0.01, ****p* < 0.001, *n* = 5); ns, not significant. EV, empty vector.

**Supplementary Fig. S11** Pharmacological effect of Amcasertib as established in SH-SY5Y (C/T) and SK-N-AS (C/C) cell models of SCA3

**a** Cell viability assays of HEK293T cells after treatment with different concentrations of Amcasertib for varying durations. Treatment with 0.5 or 1 µM Amcasertib for 48 h did not affect HEK293T viability. Similarly, SH-SY5Y, U87-MG, and SK-N-AS cells showed no change in viability under the same treatment conditions, indicating the safety of the treatment regimen. *n* = 1. **b** and **d** Western blot analysis of expanded ataxin-3 and KPNA3 in SH-SY5Y (C/T) and SK-N-AS (C/C) cells transfected with V5-Atx3 148Q following Amcasertib treatment. Amcasertib did not influence soluble expanded ataxin-3 levels in either cell line. In SK-N-AS (C/C) cells, Amcasertib reduced KPNA3 protein levels, whereas no effect was observed in SH-SY5Y (C/T) cells. GAPDH served as loading control. **c** and **e** Quantification of ataxin-3 and KPNA3 levels from **b** and **d**, normalized to GAPDH. Data are presented as means ± SEM; one-sample *t* and Wilcoxon test (**p* < 0.05, *n* = 4); ns, not significant. **f** and **h** Filter retardation assays detecting SDS-insoluble ataxin-3 levels in SH-SY5Y (C/T) and SK-N-AS (C/C) cells expressing V5-Atx3 148Q under 0, 0.5, or 1 µM Amcasertib treatment. The levels of ataxin-3 aggregates decreased in SK-N-AS (C/C) cells following treatment with 1 µM Amcasertib, but not in SH-SY5Y (C/T) cells. SDS-insoluble protein species were detected using ataxin-3 antibody. **g** and **i** Quantification of ataxin-3 aggregate levels from **f** and **h**. Data are presented as means ± SEM; one-sample *t* and Wilcoxon test (**p* < 0.05, *n* = 6); ns, not significant. **j** and **l** Fluorescence microscopy showing aggregates (highlighted by arrowheads) in SH-SY5Y (C/T) and SK-N-AS (C/C) cells expressing GFP-Atx3 148Q under 0, 0.5, or 1 µM Amcasertib treatment. Aggregate frequency was calculated as the percentage of GFP-positive cells containing ataxin-3 aggregates relative to the total number of GFP-positive cells. Scale bar, 20 μm. **k** and **m** Quantification of ataxin-3 aggregates from **j** and **l**, expressed as the percentage of aggregate-positive cells in GFP-positive cells. *n* = 1.

**Supplementary tables**

**Supplementary Table S1.** PCR reaction mixture

| 5×OneTaq buffer | 4.0 µl |
| --- | --- |
| 2 mM dNTP | 1.6 µl |
| Forward primer (10 pmol/µl) | 0.8 µl |
| Reverse primer (10 pmol/µl) | 0.8 µl |
| OneTaq polymerase | 0.125 µl |
| Betain | 4.0 µl |
| H_2_O | 7.7 µl |
| DNA | 1.0 µl |
| **Total** | **20.0 µl** |

**Supplementary Table S2.** PCR cycling protocol

| Initiation | 1 × 1 min 94°C |
| --- | --- |
| Denaturation  Annealing  Amplification | 1 min 94°C  35 × 1 min 64°C  1 min 68°C |
| Final elongation | 1 × 6 min 68°C |

**Supplementary Table S3.** Identification of candidate transcription factors potentially altered by SNP rs11618521

| **Databases** | **URL** | **TFs output** | **Experimental/Predicted** |
| --- | --- | --- | --- |
| PROMO | https://alggen.lsi.upc.es | CEBPA, CEBPB, FOXP3 | Predicted |
| PERFECTOS-APE | https://opera.autosome.org/perfectosape | SOX5, SOX10, SOX17, TFAP2A | Predicted |
| HumanTFDB | http://bioinfo.life.hust.edu.cn/HumanTFDB#!/tfbs_predict | SOX2, NANOG | Predicted |
| AliBaba2.1 | http://gene-regulation.com/pub/programs/alibaba2/ | SOX5, CEBPA | Predicted |
| Cistrome Data Browser | http://cistrome.org/db/#/ | GATA1 | Experimental |
| GTRD | https://gtrd.biouml.org/#! | PPARG | Experimental |
| ChIP-Atlas | https://chip-atlas.org/ | GATA1 | Experimental |
| VannoPortal | http://www.mulinlab.org/vportal/index.html | E2F1, CTCF | Experimental |

Potential transcription factors (TFs) altered by the rs11618521 locus were predicted by inputting two 20-bp allele-specific sequences (10 bp upstream and 10 bp downstream of the SNP) into the prediction databases, and TFs which were predicted to bind differently to the sequences harboring rs11618521 (C/T) were selected out. Chromatin immunoprecipitation sequencing (ChIP-seq) databases were further searched for TFs bound at or near the rs11618521 region.

Note: Databases were accessed and raw data were retrieved in 2023. PROMO and HumanTFDB are currently inaccessible at the time of manuscript submission.

**Supplementary Table S4.** Transcription factor expression constructs used in this study

| **Recombinant DNA** | **Source** | **Identifier** |
| --- | --- | --- |
| Flag-FOXP3 | Addgene, gift from Stefan Koch [6] | Addgene_153147 |
| hGATA-1 WT | Addgene, gift from Lea Sistonen [4] | Addgene_118352 |
| MAC_N_SOX5 | Addgene, gift from Markku Varjosalo [3] | Addgene_187742 |
| MAC_N_SOX10 | Addgene, gift from Markku Varjosalo [3] | Addgene_167791 |
| MAC_N_SOX17 | Addgene, gift from Markku Varjosalo [3] | Addgene_167793 |
| pAcGFP-Hyg-CTCF-N1 | Addgene, gift from Mark Minden [9] | Addgene_131799 |
| pcDNA3 Flag C/EBPa | Addgene, gift from Christopher Vakoc [7] | Addgene_66978 |
| pcDNA3.1-Nanog | Addgene, gift from Linzhao Cheng [1] | Addgene_28221 |
| pcDNA3.3_SOX2 | Addgene, gift from Derrick Rossi [10] | Addgene_26817 |
| pCMV-FLAG LAP2 | Addgene, gift from Joan Massague [2] | Addgene_15738 |
| pCMVHA E2F1 | Addgene, gift from Kristian Helin [5] | Addgene_24225 |
| pGL3-basic | Promega | E175A |
| pRL- CMV | Promega | E2261 |
| pSV Sport PPAR gamma 1 | Addgene, gift from Bruce Spiegelman [8] | Addgene_8886 |
| SP (RSV) AP-2 | Addgene, gift from Robert Tjian [11] | Addgene_12100 |

**Supplementary Table S5.** Antibodies used in this study

| **Antibodies** | **Source** | **Identifier (RRID)** |
| --- | --- | --- |
| Anti-Ataxin-3 | Merck (MAB5360) | AB_2129339 |
| Anti-CEBPB | Proteintech (23431-1-AP) | AB_2879278 |
| Anti-FOXP3 | Proteintech (22228-1-AP) | AB_11182376 |
| Anti-GAPDH | Santa Cruz (sc-47724) | AB_627678 |
| Anti-GATA1 | Proteintech (60011-1-Ig) | AB_2294437 |
| Anti-GFP | Santa Cruz (sc-9996) | AB_627695 |
| Anti-6×His | Proteintech (66005-1-Ig) | AB_11232599 |
| Anti-KPNA3 | Proteintech (67892-1-Ig) | AB_2918648 |
| Anti-KPNA3 | Abcam (S0386) | AB_10639243 |
| Anti-KPNA3 | Abcam (ab6038) | AB_305247 |
| Anti-Luciferase | Merck (L0159) | AB_260379 |
| Anti-NANOG | Proteintech (14295-1-AP) | AB_1607719 |
| Goat pAb to Rabbit | Abcam (ab97051) | AB_10679369 |
| IRDye 680LT Goat anti-Mouse | LI-COR (926-68020) | AB_10706161 |
| IRDye 800CW Goat anti-Mouse | LI-COR (926-32210) | AB_621842 |
| IRDye 680LT Goat anti-Rabbit | LI-COR (926-68021) | AB_10706309 |
| IRDye 800CW Goat anti-Rabbit | LI-COR (926-32211) | AB_621843 |
| Normal Rabbit IgG | Cell Signaling Technology (2729S) | AB_1031062 |

**Supplementary references**

1	Chou B-K, Mali P, Huang X, Ye Z, Dowey SN, Resar LM, Zou C, Zhang YA, Tong J, Cheng L (2011) Efficient human iPS cell derivation by a non-integrating plasmid from blood cells with unique epigenetic and gene expression signatures. Cell Res 21: 518-529 Doi 10.1038/cr.2011.12

2	Gomis RR, Alarcón C, Nadal C, Van Poznak C, Massagué J (2006) C/EBPbeta at the core of the TGFbeta cytostatic response and its evasion in metastatic breast cancer cells. Cancer Cell 10: 203-214

3	Göös H, Kinnunen M, Salokas K, Tan Z, Liu X, Yadav L, Zhang Q, Wei G-H, Varjosalo M (2022) Human transcription factor protein interaction networks. Nat Commun 13: 766 Doi 10.1038/s41467-022-28341-5

4	Hietakangas V, Anckar J, Blomster HA, Fujimoto M, Palvimo JJ, Nakai A, Sistonen L (2005) PDSM, a motif for phosphorylation-dependent SUMO modification. Proc Natl Acad Sci U S A 103: 45-50

5	Lukas J, Petersen BO, Holm K, Bartek J, Helin K (1996) Deregulated expression of E2F family members induces S-phase entry and overcomes p16INK4A-mediated growth suppression. Mol Cell Biol 16: 1047-1057

6	Moparthi L, Koch S (2020) A uniform expression library for the exploration of FOX transcription factor biology. Differentiation 115: 30-36 Doi 10.1016/j.diff.2020.08.002

7	Roe J-S, Mercan F, Rivera K, Pappin DJ, Vakoc CR (2015) BET Bromodomain Inhibition Suppresses the Function of Hematopoietic Transcription Factors in Acute Myeloid Leukemia. Mol Cell 58: 1028-1039 Doi 10.1016/j.molcel.2015.04.011

8	Tontonoz P, Hu E, Graves RA, Budavari AI, Spiegelman BM (1994) mPPAR gamma 2: tissue-specific regulator of an adipocyte enhancer. Genes Dev 8: 1224-1234

9	Wang AJ, Han Y, Jia N, Chen P, Minden MD (2019) NPM1c impedes CTCF functions through cytoplasmic mislocalization in acute myeloid leukemia. Leukemia 34: 1278-1290 Doi 10.1038/s41375-019-0681-8

10	Warren L, Manos PD, Ahfeldt T, Loh Y-H, Li H, Lau F, Ebina W, Mandal PK, Smith ZD, Meissner Aet al (2010) Highly efficient reprogramming to pluripotency and directed differentiation of human cells with synthetic modified mRNA. Cell Stem Cell 7: 618-630 Doi 10.1016/j.stem.2010.08.012

11	Williams T, Tjian R (1991) Analysis of the DNA-binding and activation properties of the human transcription factor AP-2. Genes Dev 5: 670-682

**Supplemental notes**

**The EUROSCA Network collaborators and their affiliations**

Peter Bauer^1,2,3^, José Berciano^4^, Sylvia Boesch^5^, Alexis Brice^6^, Alexandra Durr^6^, Sylvie Forlani^6^, Paola Giunti^7^, Heike Jacobi^8^, Thomas Klockgether^9^, Bela Melegh^10^, Massimo Pandolfo^11^, Olaf Riess^1^, Tanja Schmitz-Hübsch^12,13,14,15^, Ludger Schöls^16,17^, Jörg B. Schulz^18^, Giovanni Stevanin^6,19^, Sandra Szymanski^20^, Sophie Tezenas du Montcel^6^, Dagmar Timmann^21^, Bart P. C. van de Warrenburg^22^

^1^ Institute of Medical Genetics and Applied Genomics, University of Tübingen, Tübingen, Germany

^2^ Centogene GmbH, 18055 Rostock, Germany

^3^ Pomeranian Medical University PUM, Szczecin, Poland

^4^ Servicio Cántabro de Salud Santander, University Hospital Marqués de Valdecilla (IDIVAL), University of Cantabria (UC), Santander, Spain

^5^ Department of Neurology, Medical University Innsbruck, Innsbruck, Austria

^6^ Sorbonne Université, Institut du Cerveau - Paris Brain Institute - ICM, Inserm, CNRS, APHP, University Hospital Pitié-Salpêtrière, Paris, France

^7^ Department of Molecular Neuroscience, Institute of Neurology, University College London, London, United Kingdom

^8^ Department of Neurology, University Hospital Heidelberg, Heidelberg, Germany

^9^ German Center for Neurodegenerative Diseases (DZNE), Bonn, Germany

^10^ Department of Medical Genetics, and Szentagothai Research Center, University of Pécs, Pécs, Hungary; Department of Neurology, Zala County Hospital, Zalaegerszeg, Hungary

^11^ Université Libre de Bruxelles (ULB), Neurology Service, ULB Hôpital Erasme, ULB Laboratory of Experimental Neurology, Brussels, Belgium

^12^ Experimental and Clinical Research Center, a cooperation between the Max Delbrück Center for Molecular Medicine in the Helmholtz Association and Charité Universitätsmedizin, Berlin, Germany

^13^ Experimental and Clinical Research Center, Charité-Universitätsmedizin Berlin, corporate member of Freie Universität Berlin and Humboldt-Universität zu Berlin, Berlin, Germany

^14^ Max Delbrück Center for Molecular Medicine in the Helmholtz Association, Berlin, Germany

^15^ Charité - Universitätsmedizin Berlin, corporate member of Freie Universität Berlin, Humboldt-Universität zu Berlin, and Berlin Institute of Health, NeuroCure Clinical Research Center, Berlin, Germany

^16^ Department of Neurodegeneration and Hertie-Institute for Clinical Brain Research, University of Tübingen, Tübingen, Germany

^17^ German Center for Neurodegenerative Diseases (DZNE), Tübingen, Germany

^18^ Department of Neurology, RWTH Aachen University, Aachen, Germany; JARA-Translational Brain Medicine, Aachen-Jülich, Aachen, Germany

^19^ Université de Bordeaux - CNRS-Centre National de la Recherche Scientifique, INCIA-Institut de Neurosciences Cognitives et Intégratives d'Aquitaine, UMR 5287, Bordeaux, France

^20^ Neurology Bochum, Ruhr University Bochum, Bochum, Germany

^21^ Department of Neurology, Essen University Hospital, University of Duisburg-Essen, Essen, Germany

^22^ Department of Neurology, Donders Institute for Brain, Cognition and Behaviour, Radboud University Medical Center, Nijmegen, Netherlands
